# Recessive Effect of *GC-NPFFR2* rs137147462 on Somatic Cell Score (Mastitis Susceptibility) in Japanese Holsteins

**DOI:** 10.3390/ani15223239

**Published:** 2025-11-08

**Authors:** Yoshiyuki Akiyama, Takaaki Ando, Nobuhiro Nozaki, Mohammad Arif, Yutaro Ide, Shaohsu Wang, Naoki Miura

**Affiliations:** 1 Joint Graduate School of Veterinary Medicine, Kagoshima University, Kagoshima 890-0065, Japan; 2 South Kyushu Livestock Veterinary Center, Joint Faculty of Veterinary Medicine, Kagoshima University, Soo 899-4101, Japan; 3 Department of Microbiology and Hygiene, Bangladesh Agricultural University, Mymensingh 2202, Bangladesh; 4 Veterinary Teaching Hospital, Joint Faculty of Veterinary Medicine, Kagoshima University, Kagoshima 890-0065, Japan

**Keywords:** dairy cattle, Holstein, mastitis, somatic cell score, *GC-NPFFR2*, *DGAT1*, p.K232A, *BRCA1*

## Abstract

**Simple Summary:**

Dairy cows frequently suffer from mastitis, or inflammation of the udder, and that causes major economic loss to farmers. If farmers could breed cows with better natural resistance to mastitis, they could thus achieve an important goal for sustainable milk production. In this study, we investigated data on genetics and milk from Japanese Holstein cows over a ten-year period, to see if we could identify any links in the data related to udder health. We found cows with one particular genetic variation were statistically at much greater risk of developing mastitis. This information on dairy cow genetics may help cattle breeders raise healthier, and more productive herds.

**Abstract:**

We evaluated four candidate SNPs (*GC-NPFFR2* rs137147462, *GC-NPFFR2* rs109452259, *BRCA1* rs134817801, and *DGAT1* p.K232A) previously reported in relation to mastitis or milk production traits, using 10,729 test-day phenotypic records collected over 10 years from 269 Japanese Holstein cows (*Bos taurus*) enrolled in the national Dairy Herd Improvement (DHI) program. Linear mixed models were used to estimate genotypic effects on somatic cell score (SCS) and to test multiple inheritance models. To assess clinical relevance, mastitis severity was further analyzed using categories defined by somatic cell counts (SCC). Among the SNPs tested, *GC-NPFFR2* rs137147462 showed the clearest and most consistent association with SCS under a recessive model, with GG cows exhibiting higher SCS throughout lactation. Ordinal logistic regression confirmed a higher probability of progression to severe mastitis in GG cows. *DGAT1* p.K232A showed additive effects, with the A allele increasing milk yield while lowering fat and protein percentages. AA cows also showed higher SCS under a modest recessive effect. *BRCA1* rs134817801 and *GC-NPFFR2* rs109452259 had minimal effects. These findings support *GC-NPFFR2* rs137147462 as a promising marker for mastitis resistance and indicate the importance of considering not only additive but also recessive genetic models in genomic selection strategies.

## 1. Introduction

Bovine mastitis presents a major challenge for the dairy industry, as it leads to decreased milk yield, discarded milk, higher veterinary costs, and increased culling [1,2,3]. It is a complex condition to which environmental factors, herd management practices, and genetic predisposition all may contribute [4,5]. Losses to farmers may be substantial whether the mastitis is clinical or subclinical. Cases of the latter are particularly difficult to detect, although subclinical mastitis can reportedly be responsible for elevated bulk-tank somatic cell count (SCC) and degraded milk quality [1,2,5]. As well as causing economic losses, mastitis has an adverse impact on animal welfare and drives increased antimicrobial use at the herd level, making it an important challenge for the sustainability of dairy production [3,5,6,7].

Genetic variation in mastitis resistance is well established, although the heritability of clinical mastitis is generally low, ranging from 0.01 to 0.10 [8,9]. In contrast, somatic cell score (SCS), which is the logarithmic transformation of SCC, exhibits moderate heritability (0.15 to 0.20), and is widely used as an indicator trait for improving udder health through selective breeding [10]. SCCs are routinely recorded in testing under the Japanese national Dairy Herd Improvement (DHI) program, and reflect leukocyte infiltration during intramammary infection. As base-2 logarithmic transformation stabilizes any variance present in SCC data, and yields an approximately normal distribution of data for genetic evaluation, SCS has been widely adopted as a proxy indicator for udder health and predictions of milk loss and increased risk of clinical mastitis [5,10,11,12,13,14]. However, despite the success of genomic selection for production traits, the identification of robust and biologically relevant markers for mastitis resistance remains a challenge as shown in previous studies including *TLR4* [15,16]. Single nucleotide polymorphisms (SNPs) represent a potentially fruitful line of research for establishing such genetic markers.

The *GC-NPFFR2* region on BTA6 has been identified as a major source of signals associated with SCS in a large-scale, genome-wide association study (GWAS) in North American Holsteins [17]. That study involved two complementary approaches: one with an approximate generalized least squares (AGLS) method, and the other with a Bayesian linear mixed model implemented via BOLT-LMM (version 2.3.2) software [18,19]. *GC* encodes the vitamin D–binding protein, while *NPFFR2* encodes a neuropeptide receptor implicated in neuro-immune and inflammatory regulation [20,21,22,23]. Within the *GC-NPFFR2* region, the rs137147462 and rs109452259 SNPs appear to be the most promising for further investigation. Accordingly, we opted to target them for validation as independent genetic markers for SCS (and hence mastitis) in a Japanese Holstein population. We further opted to evaluate *BRCA1* rs134817801 as a useful comparator SNP, because of its essential roles in DNA repair and immune function and its previously reported association with SCS in cattle [24]. We also targeted *DGAT1* as a major quantitative trait locus (QTL) for milk fat synthesis, focusing on the well-known causal SNP (p.K232A) in that QTL. *DGAT1* p.K232A is known to exert a strong effect on milk composition [25,26,27].

Against this background, in the current study, we aimed to evaluate the effects of the four candidate SNPs (*GC-NPFFR2* rs137147462, *GC-NPFFR2* rs109452259, *BRCA1* rs134817801, and *DGAT1* p.K232A) initially identified for their potential association with SCS, on test-day traits in Japanese Holstein cows. Using longitudinal records from the Japanese national DHI program, we investigated the associations of the four candidate SNPs with SCS and major milk composition traits. The two analytical frameworks we applied were a main-effect model, and an interaction model including genotype-by-lactation-stage effects. This approach was designed to identify reliable genetic markers for mastitis resistance and to determine whether the potential effects of these SNPs are consistent or changeable across different stages of lactation. Although this study provides detailed longitudinal insights, it was conducted using records from a single commercial herd, and the number of cows carrying certain rare genotypes was limited; therefore, the findings should be interpreted within this population context.

## 2. Materials and Methods

### 2.1. Animals and Phenotypic Records

Data were obtained from a commercial Holstein dairy farm in Japan participating in the national DHI program. All milk testing was conducted by the Hokkaido Dairy Milk Recording & Testing Association (Sapporo, Japan) under this program. SCC and milk composition were analyzed using a CombiFoss 7 or FT+ (Foss, Hillerød, Denmark). Milk samples were collected once daily from individual cows during either the morning or evening milking on each test day. A total of 10,729 test-day records from 269 cows, collected between 2010 and 2025, were analyzed. Records included milk yield, fat content (fat%), protein content (protein%), solids-not-fat content (SNF%), and SCC, which was converted to SCS for evaluation [12]. Each record was linked to cow identity, parity, and days in milk (DIM) for use in longitudinal models. Ear tissue samples were collected during routine ear tagging carried out in accordance with Japan’s national cattle identification system, and animals were subject to no experimental procedures; therefore, ethical approval was waived for this study. The 269 cows in the current study population included 256 individuals we had targeted in a previous, separate evaluation of linkage disequilibrium for another SNP [28].

### 2.2. DNA Extraction and Genotyping

Genomic DNA was extracted with the GeneJET Genomic DNA Purification Kit (Thermo Fisher Scientific, Waltham, MA, USA). Four SNPs (*GC*-*NPFFR2* rs137147462, *GC*-*NPFFR2* rs109452259, *BRCA1* rs134817801, and *DGAT1* p.K232A) were genotyped with Custom TaqMan assays on a StepOne Plus Real-Time PCR System (Thermo Fisher Scientific, Waltham, MA, USA). Genotype classification followed the ARS-UCD2.0 reference genome, with details summarized in Table 1.

### 2.3. Evaluation of Genotypic Distributions

We evaluated Hardy–Weinberg equilibrium (HWE) and pairwise linkage disequilibrium (r^2^ and D′) across the four loci and computed exact 95% confidence intervals for allele frequencies with the Clopper–Pearson method, using R software (version 4.3).

### 2.4. Data Classification

Test-day records were classified into categories of DIM stage, parity, and season, and calving year. Detailed class definitions and descriptive statistics for all traits are provided in Supplementary Table S1.

### 2.5. Statistical Analysis

All primary analyses were conducted under a codominant framework, in which the three genotype classes (Wild, Heterozygote, Mutant) were treated as distinct fixed levels. For each SNP, two linear mixed models were constructed: the first estimated the main effect of genotype, and the second included genotype × DIM stage interactions to evaluate lactation-stage specific effects. In both models, the response variable was one of the test-day traits: milk yield, fat percentage, protein percentage, SNF percentage, or SCS. Fixed effects were DIM stage, parity, season, and calving year, and cow identity was included as a random effect to account for repeated measures. The models were specified as:

Main effect model:
  (1) $$y_{ijkltmn}=\mu +G_{i}+D_{j}+P_{k}+S_{l}+{CY}_{t}\;{+\;a}_{m}+\varepsilon_{ijkltmn}$$
Interaction model:
  (2) $$y_{ijkltmn}=\mu +G_{i}+D_{j}+(G_{i}\times {D_{j})+P}_{k}+S_{l}+{CY}_{t}\;+a_{m}+\varepsilon_{ijkltmn}$$
  where $y_{ijkltmn}$ represents the test-day phenotype, $G_{i}$ is the fixed effect of genotype, $D_{j}$ is DIM stages, $G_{i}\times D_{j}$ is the genotype-by-DIM interaction, $P_{k}$ is parity group, $S_{l}$ is season, ${CY}_{t}$ is calving year, $a_{m}$ is the random effect of cowID and *n* denotes repeated test-day records within each cow, and $\varepsilon_{ijkltmn}$ is the residual error.

Least-squares means (LSMs) were estimated for each genotype, and multiple comparisons were adjusted using Tukey’s honestly significant difference (HSD) test at α = 0.05. Genotype effects were evaluated by Type III ANOVA, with significance levels denoted as * *p* < 0.05, ** *p* < 0.01, and *** *p* < 0.001. For the main-effect model, SNPs showing significant genotype effects were additionally subjected to false discovery rate (FDR) correction across SNP–trait combinations to confirm the robustness of the results. FDR correction was applied only within the subset of SNP–trait combinations that showed significant genotype effects in the main-effect codominant model, rather than across all tests performed.

In addition to the codominant framework, additive, dominant, recessive, and overdominant inheritance models were also evaluated for each SNP–trait combination. For a bi-allelic locus with alleles A (wild-type) and B (mutant) (as defined in Table 1), the codominant model treated genotype as a categorical variable with three levels (AA vs. AB vs. BB), representing each genotype separately; additive used B-allele dosage (AA = 0, AB = 1, BB = 2); dominant compared AA vs. AB+BB; recessive compared AA+AB vs. BB; and overdominant compared AB vs. AA+BB. Genotypes were coded numerically as (0/1/2 for additive; 0/1 for dominant, recessive, or overdominant), with definitions consistent with standard practice. The same fixed-and-random-effects structure was applied across all models.

To complement the mixed-model analyses, a simplified auxiliary analysis was performed to appraise clinical relevance based on the highest SCC during each lactation. An ordinal logistic regression was fitted for mastitis severity, defined as corresponding to healthy cattle (<200,000 cells/mL), subclinical cases (≥200,000 and <400,000), or clinical cases (≥400,000). Genotypes were modeled as categorical variables with AA as the reference. The proportional-odds assumption was evaluated with the Brant test and was not violated (Omnibus *p* = 0.388). These SCC cut-offs followed established individual-cow criteria and recent practice in udder-health research [29,30,31].

### 2.6. Software

All statistical analyses were performed in R (version 4.3) [32] using HardyWeinberg (version 1.7.8) for HWE tests [33], genetics (version 1.3.8.13) for LD analyses [34], lme4 (version 1.1-37) and lmerTest (version 3.1-3)for linear mixed models [35,36], emmeans (version 1.11.2) for least-squares means [37], MASS (version 7.3-65) for ordinal logistic regression [38], and broom (version 1.0.9) and brant (version 0.3.0) for regression diagnostics [39]. Figures were generated with ggplot2 (version 3.4.2) [40].

## 3. Results

### 3.1. Genetic Variation and Independence of Candidate SNPs

All four loci were polymorphic with adequate minor allele frequencies. Exact 95% confidence intervals for allele frequencies are included in Table 2. Pairwise LD across loci was weak, justifying separate analyses (Table 3).

### 3.2. Main Effects of Genotype on Test-Day Milk Production and Somatic Cell Score

**Figure 1:**
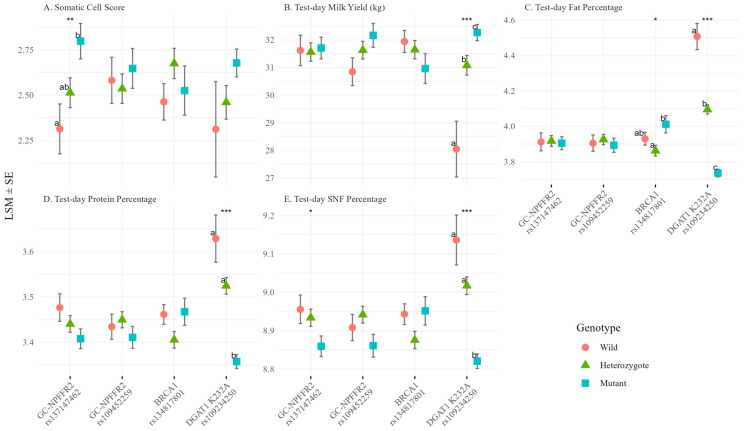
Genotype effects on test-day traits. (**A**) Somatic Cell Score, (**B**) Test-day Milk Yield (kg), (**C**) Test-day Fat Percentage, (**D**) Test-day Protein Percentage, and (**E**) Test-day Solids-not-fat (SNF) Percentages are shown for four candidate SNPs. Least squares means (LSMs ± SE) were estimated using linear mixed models including DIM stage, parity group, and season as fixed effects and cow ID as a random effect. For genotype main effects, significance was assessed by ANOVA (* *p* < 0.05; ** *p* < 0.01, *** *p* < 0.001). Different letters indicate statistically significant differences between genotypes (Tukey’s HSD test, *p* < 0.05).

SCS was significantly affected only by *GC-NPFFR2* rs137147462, among the four candidate SNPs (*p* = 0.0099), with GG cows consistently exhibiting the highest values; Tukey’s HSD showed a significant difference between GG and AA, with AG intermediate (Figure 1; Supplementary Table S2). The overall SNP effect remained significant after FDR correction (FDR-adjusted *p* = 0.040), confirming its robustness. For the same SNP, SNF% showed a small but significant overall difference (*p* = 0.047), although it became non-significant after FDR correction (FDR-adjusted *p* = 0.14), and pairwise contrasts were not significant. Four production traits (milk yield, fat%, protein%, and SNF) were significantly affected by *DGAT1* p.K232A (all *p* < 0.001; FDR-adjusted *p* < 0.001), reproducing the established trade-off between lower yield and higher component content in KK cows. *BRCA1* rs134817801 was associated with fat% (*p* = 0.0296), but this association became non-significant after FDR correction (FDR-adjusted *p* = 0.099). CC cows had the highest values, and similar non-additive tendencies were observed, for protein% (*p* = 0.073) and SNF% (*p* = 0.079), and AC cows showed lower values than both homozygotes for these parameters. *GC-NPFFR2* rs109452259 showed no significant effects on any trait.

### 3.3. Genotype-by-DIM Interactions Affecting Milk Traits and SCS

**Figure 2:**
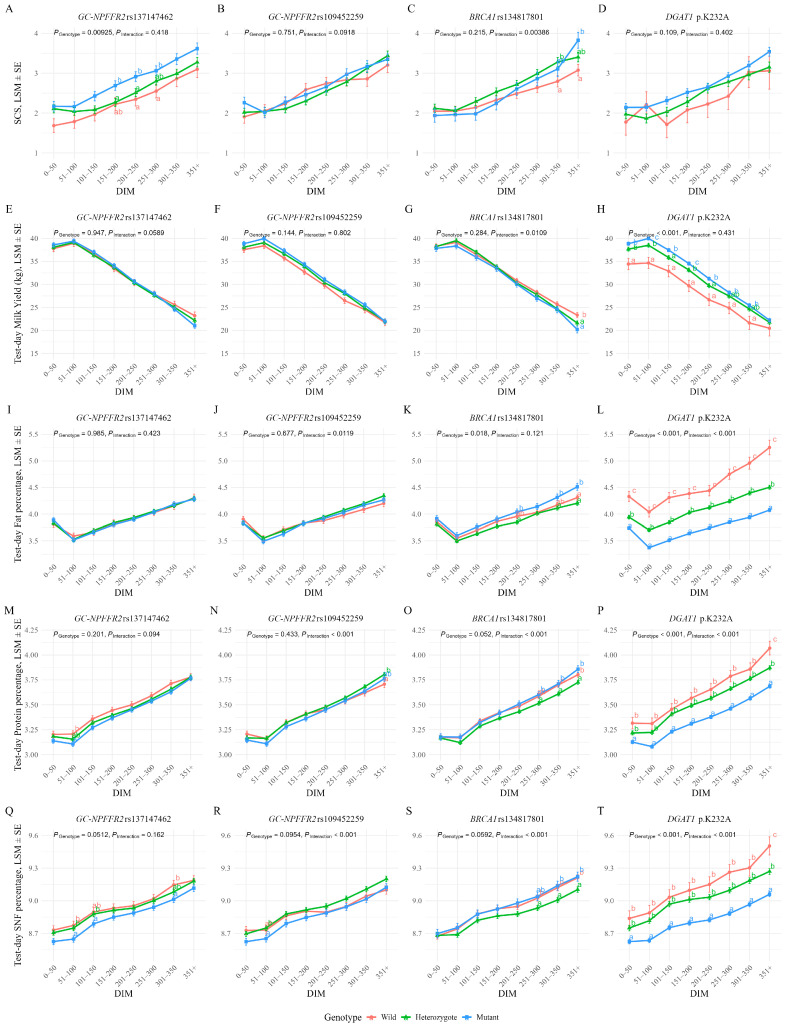
LSM ± SE of test-day SCS and milk production traits across lactation stages by genotype. Panels (**A**–**D**): SCS; (**E**–**H**): milk yield; (**I**–**L**): fat%; (**M**–**P**): protein%; (**Q**–**T**): SNF. Each row corresponds to one SNP: *GC-NPFFR2* rs137147462 (**A**,**E**,**I**,**M**,**Q**), *GC-NPFFR2* rs109452259 (**B**,**F**,**J**,**N**,**R**), *BRCA1* rs134817801 (**C**,**G**,**K**,**O**,**S**), and *DGAT1* p.K232A (**D**,**H**,**L**,**P**,**T**). DIM were grouped by 50-day intervals up to 350 d, with recorded values after 350 d combined. Different letters indicate statistically significant differences between genotypes (Tukey’s HSD test, *p* < 0.05).

Results are summarized in Figure 2 (multi-panel A–T) and Supplementary Table S3.

#### 3.3.1. *GC*-*NPFFR2* rs137147462

Cows with the GG genotype consistently showed higher SCS throughout lactation (overall *p* = 0.00925). At 151–200 DIM and 201–250 DIM, GG cows had significantly higher SCS than AG cows, while AA cows showed intermediate values (Tukey, *p* < 0.05; Figure 2A). A similar pattern was observed at 251–300 DIM, where GG cows showed significantly higher SCS than AA cows. These results provide supporting evidence for a recessive effect of the G allele. This SNP showed no significant main effects on protein% or SNF%, but the order of mean values by genotype (AA > AG > GG) remained stable throughout lactation. For protein%, the overall effect was not significant (*p* = 0.201), but AA cows tended to have higher values than GG cows between 51 and 150 DIM, with AG cows showing intermediate values. For SNF%, the overall effect approached significance (*p* = 0.0512). At 51–100 DIM, both AA and AG cows showed significantly higher SNF than GG cows (*p* < 0.05), and similar trends were observed at 101–150 DIM and 301–350 DIM. Taken together, these results indicate that although effect sizes were small, the descending order of AA > AG > GG was consistently maintained across lactation for both protein% and SNF% (Figure 2M,Q).

#### 3.3.2. *GC*-*NPFFR2* rs109452259

No significant effects were observed on SCS, but significant genotype-by-DIM interactions occurred for fat% (*p* = 0.0119), protein% (*p* < 0.001), and SNF% (*p* < 0.001). CC cows showed a marked decline in these three parameters from 201 to 250 DIM.

For protein%, significant pairwise differences were detected only in late lactation (351 DIM and after), where CA cows showed the lowest values, while AA and CC cows showed higher levels. For SNF%, significant differences were observed only at 51–100 DIM, with CA cows showing significantly lower values than AA and CC cows (*p* < 0.05; Figure 2J,N,R).

#### 3.3.3. *BRCA1* rs134817801

We detected no main SNP effect on SCS, but a significant interaction was detected (overall *p* = 0.00386). AC cows generally showed higher SCS than AA cows, while CC cows showed a sharply rising SCS from 150 DIM and their SCS exceeded that in AA cows in late lactation. At 301–350 DIM, AC cows showed a significantly higher SCS than AA cows, and CC cows showed a significantly higher SCS than AA cows at 351 DIM and after (*p* < 0.05; Figure 2C). For milk yield, AA cows had significantly higher values than AC and CC cows in late lactation (351 DIM and after; *p* < 0.05). CC cows consistently showed the highest fat%, protein%, and SNF%, with differences becoming more pronounced in mid-to-late stages of lactation (Figure 2G,K,O,S).

#### 3.3.4. *DGAT1* p.K232A

No significant effects on SCS were observed, but strong effects were found for other production traits (all *p* < 0.001). Milk yield descended (by genotype) in the order of AA > KA > KK, whereas fat%, protein%, and SNF% all showed values in a descending order of KK > KA > AA. Fat% showed significant pairwise differences between each of the three genotypes at every stage of lactation (*p* < 0.05; Figure 2L), and protein% and SNF%, differed significantly between the three genotypes at 351 DIM and after (*p* < 0.05; Figure 2P,T).

### 3.4. Evaluation of Genetic Inheritance Models

Four inheritance models (additive, dominant, recessive, and overdominant) were compared for each SNP–trait combination. The results for SCS evaluations, with significant effects (*p* < 0.05), are summarized in Table 4, and the comprehensive results for all traits are provided in Supplementary Table S4.

In these inheritance models, SCS was significantly affected by *GC-NPFFR2* rs137147462 and *DGAT1* p.K232A. For *GC-NPFFR2* rs137147462, significant effects were detected under both the dominant (*p* = 0.037) and recessive (*p* = 0.0054) models, with the latter showing the strongest association; GG cows consistently exhibited higher SCS values than AA or AG cows. *DGAT1* p.K232A also showed a significant recessive effect (*p* = 0.044), with AA cows having slightly higher SCS than KA and KK cows.

These findings support a predominantly recessive mode of inheritance for *GC-NPFFR2* rs137147462 and indicate that the *DGAT1* locus exerted a minor but statistically significant effect on SCS, whereas other loci showed no meaningful associations.

### 3.5. Supporting Analysis of SCC-Based Mastitis Severity Associated with GC-NPFFR2 rs137147462

Given that *GC-NPFFR2* rs137147462 showed significant associations with SCS in multiple models, we further examined its clinical relevance using ordinal logistic regression with mastitis severity classified as healthy, subclinical mastitis (SCM), or clinical mastitis (CM). GG cows were likely to show a progression to more severe mastitis than AA cows (OR = 1.63; 95% CI 1.18–2.28; *p* = 0.0035), whereas AG cows showed no significance (Table 5). The proportional-odds assumption was not violated (Brant omnibus *p* = 0.39).

## 4. Discussion

### 4.1. Effects of Two Loci in the GC-NPFFR2 Region on SCS and SCC-Based Mastitis Traits

We targeted two SNPs in the *GC-NPFFR2* region on BTA6 (rs137147462 and rs109452259) for investigation in this study, based on significant signals reported in a large GWAS in North American Holsteins [17]. In our study population, *GC-NPFFR2* rs137147462 showed a robust association with SCS, detected primarily under a recessive model (*p* = 0.0054), with weaker support under a dominant model (*p* = 0.037). Furthermore, GG cows showed consistently higher SCS values than AA or AG cows (overall *p* = 0.00925). Mastitis-severity analyses provided further evidence for this recessive pattern, which occurred without clear penalties on milk yield, fat%, or protein%; however, we noted a non-significant trend toward lower SNF%. Within the DIM-adjusted framework, the GG genotype showed persistently higher SCS values across the lactation period, with differences versus other genotypes reaching significance at 151–200 and 201–250 DIM (vs. AG) and again at 251–300 DIM (vs. AA). These results indicate a persistent recessive effect, rather than a stage-restricted fluctuation. For milk-composition traits, genotype rankings were small and stable across stages. Milk lactose percentage is negatively associated with SCC in dairy cattle and declines during or after intramammary inflammation; similar patterns are reported in buffaloes and small ruminants [41,42,43,44]. Because lactose is the principal milk osmole, epithelial damage and leaky tight junctions reduce the amount of lactose retained in the alveolar lumen, leading to lower milk yield, and altered mineral balance (mediated by increased sodium and potassium concentrations) [45,46,47,48,49,50]. Considering this phenomenon, lactose is a promising predictor of subclinical mastitis, and genomic studies show overlap between lactose-associated regions and loci for mastitis/udder-health traits [42,51,52,53,54]. In our data, protein% did not differ consistently by genotype, whereas GG cows (the higher-SCS genotype) showed a slightly lower SNF (overall effect approaching significance). Given that lactose dominates SNF, this pattern is most plausibly explained by a modest reduction in lactose rather than protein, aligning with the established SCC–lactose antagonism [41,42].

The results for *GC-NPFFR2* rs109452259 showed a contrasting pattern. This SNP exerted no sustained effect on SCS, but it exhibited stage-dependent associations with milk composition, consistent with significant genotype-by-DIM interactions for fat%, protein%, and SNF%. Based on these findings, we suggest that rs109452259 reflects a haplotypic structure in the specific chromosomal region rather than an independent causal variant.

Although mastitis resistance is regarded as generally polygenic [15,55], our findings indicate that a single SNP, *GC-NPFFR2* rs137147462, can exert a consistent influence on SCS when non-additive inheritance is considered. Trade-offs between mastitis resistance and milk yield have previously been reported [56,57], whereas a copy number variant (CNV) in the *GC* region has been to shown reduce resistance but increase milk production [58]. Our results suggest that the effect of *GC-NPFFR2* rs137147462 may not be governed by such trade-offs, and raise the possibility that this SNP is related to structural variation or has a functional role in itself. Such a marker could play a valuable role in selective breeding, but validation in larger and more diverse populations is essential. *GC* encodes vitamin D-binding protein, regulating vitamin D metabolism and immune function [20], and *NPFFR2* is linked to anti-inflammatory macrophage activation [21,22,23].

### 4.2. Effects of BRCA1 rs134817801 on SCS and Milk Composition Traits

*BRCA1* is a tumor-suppressor gene involved in DNA repair and cell-cycle control [59,60]. In cattle, it lies on BTA19 within a QTL for SCS and milk composition traits [61,62,63], and its missense variant, rs134817801, is reportedly associated with SCS in Chinese Holsteins [24].

In our Japanese Holstein population, we detected no main *BRCA1* rs134817801 effect on SCS; however, we did detect a significant interaction effect for genotype-by-DIM, with CC cows showing sharply increasing SCS in late lactation and AC cows showing generally elevated SCS values. Milk yield also showed a stage-dependent reversal, with higher values in AA than AC or CC cows in later lactation. In contrast, this SNP had clear effects on milk composition: CC cows consistently showed the highest fat%, protein%, and SNF% values, whereas heterozygotes showed the lowest. Model comparisons supported recessive and overdominant inheritance, consistent with a pattern of heterozygote disadvantage.

Compared with *GC-NPFFR2* rs109452259, which showed heterozygote advantage for SNF%, *BRCA1* rs134817801 exhibited the opposite pattern, underscoring the diversity of non-additive effects among mastitis-associated loci. Although the additive signal of this SNP is limited and it may be overlooked in conventional GWAS, its detection through candidate-gene approaches highlights the value of longitudinal and non-additive models in uncovering hidden genetic effects.

### 4.3. Effects of DGAT1 p.K232A on Milk Composition and Mastitis-Related Traits

*DGAT1* encodes a key enzyme for triglyceride synthesis, and its p.K232A polymorphism is a well-characterized causal mutation located in a major QTL on BTA14. This variant exerts a substantial effect on milk fat content and smaller antagonistic effects on milk yield [25,64], findings that have been consistently replicated across breeds and confirmed in meta-analyses [27].

In our study population, *DGAT1* p.K232A showed a significant association with SCS under a recessive model (*p* = 0.044), with AA cows exhibiting higher SCS than KA or KK cows. Given that the K-homozygote was rare in our cohort (KK 4.8%, *n* = 13; see genotype counts in Table 2), estimates for contrasts involving KK and made in stratified analyses carry wider uncertainty and should be interpreted with caution. Our results provide interesting evidence in areas where previous reports are inconsistent: some reports have suggested favorable effects of the K allele [65,66], whereas others have found no meaningful relationship [67,68]. Taking our data and the previous reports collectively, we suggest that any link between *DGAT1* and udder health is likely minor and population specific.

In contrast, we confirmed strong additive effects on milk production traits for this SNP: KK cows produced less milk but showed higher fat, protein, and SNF percentages, patterns that intensified in later lactation. Model comparisons suggested additive inheritance for milk yield and milk fat content, and recessive effects for protein and SNF, consistent with prior meta-analyses [27].

According to the inheritance–model comparisons summarized in Supplementary Table S4, fat% followed an additive pattern, whereas both protein% and SNF% showed their most markedly statistically significant results under a recessive coding (lowest *p*-values), with AA cows being lowest for both traits. Notably, the proportional decline in SNF for AA cows appeared larger than that in protein% (visually evident in Figure 2), which is unlikely to be explained by reduced protein alone. Given that lactose constitutes the major component of SNF, this pattern may indicate a concurrent reduction in lactose, consistent with substrate reallocation under *DGAT1* p.K232A. Taking the relevant results together, we suggest that p.K232A exerted clear additive effects on fat and milk yield, while minor recessive decreases in protein and SNF were observed in AA cows. Such a phenomenon may be best interpreted as a metabolic trade-off within mammary synthesis rather than a change in udder-health status. Such an interpretation is consistent with the SCS–SNF antagonism noted earlier for *GC-NPFFR2* rs137147462.

### 4.4. Limitations, Perspectives, and Practical Implications

This study has several limitations that should be acknowledged. The sample size was moderate (269 cows), and although genotype frequencies were generally well balanced, some less frequent genotypes, particularly p.K232A KK, were underrepresented. Consequently, the statistical power to detect recessive effects was limited when the recessive homozygote class was rare, and precision further declined in stratified analyses. The dataset originated from a single commercial herd, which may limit the generalizability of the findings to wider Holstein populations. Generalizability thus needs to be confirmed with analysis beyond this single-herd setting, to confirm reproducibility in independent herds. However, in the mixed model framework, parity, season, calving year, and DIM stage were included as fixed effects, and cow identity was included as a random effect, thereby accounting for variation related to management, environment, and repeated measurements within cows. These model structures help mitigate the potential effects of farm-level management or temporal factors such as feed changes and climate fluctuations. From a broader perspective, this work provides a targeted validation of SNPs previously identified by GWAS, demonstrating that candidate variants can yield consistent phenotypic associations when evaluated under appropriate genetic models. The use of mixed models across DIM stages allowed us to detect genotype effects that would likely be masked in cross-sectional or purely additive analyses. While future research using high-density SNP arrays or whole-genome sequencing will be essential to pinpoint causal variants and resolve the surrounding haplotype structures, the present study highlights the continuing value of hypothesis-driven validation studies in bridging large-scale genomic signals with practical genetic markers. Such markers can support genomic selection strategies that improve udder health without compromising milk productivity, thereby enhancing the sustainability of dairy production.

## 5. Conclusions

In conclusion, we suggest that non-additive genetic signals relevant to udder health can be elucidated through analyses targeting longitudinal test-day records with explicit inheritance-model comparisons. Going forward, research in the near future should involve validation in larger multi-herd and international populations, and functional studies to elucidate the relevant biological mechanism. In the longer term, integrating validated markers into genomic selection programs and clarifying their physiological roles may enhance udder health and milk productivity through sustainable dairy breeding. The expected *DGAT1* p.K232A associations served as an internal positive control, supporting the robustness of our analytical framework.

## Figures and Tables

**Table 1:** Target SNPs and genotype classification.

Gene (Locus)	rsID	Position	Reference Allele (Wild-Type)	Alternate Allele (Mutant)	Assay ID
*GC-NPFFR2*	rs137147462	Chr6: 87,153,414	A	G	ANFVV7A
*GC-NPFFR2*	rs109452259	Chr6: 87,068,809	C	A	ANGZPR7
*BRCA1*	rs134817801	Chr19: 43,092,929	A	C	AN2XG4G
*DGAT1* p.K232A	rs109234250 /rs109326954	Chr14: 611,019–611,020	AA (K; Lysine)	GC (A; Alanine)	ANH6HP7

Positions are based on the ARS-UCD2.0 bovine genome assembly, with reference alleles defined as wild type.

**Table 2:** Genotype and allele frequencies with HWE test results.

		Genotypes		Alleles		*p*-Value in HWE Test
		*n*	Frequency (%) (95%CI)	*n*	Frequency (%) (95%CI)	
*GC-NPFFR2* rs137147462	AA(A)	48	17.8 (13.5–23.0)	226	42.0 (37.8–46.3)	
	AG	130	48.3 (42.2–54.5)			0.901
	GG (G)	91	33.8 (28.2–39.8)	312	58.0 (53.7–62.2)	
*GC-NPFFR2* rs109452259	CC(C)	57	21.2 (16.5–26.6)	252	46.8 (42.6–51.2)	
	CA	138	51.3 (45.2–57.4)			0.713
	AA (A)	74	27.5 (22.3–33.3)	286	53.2 (48.8–57.4)	
*BRCA1* rs134817801	AA(A)	92	34.2 (28.5–40.2)	313	58.2 (53.9–62.4)	
	AC	129	48.0 (41.9–54.1)			0.803
	CC(C)	48	17.8 (13.5–23.0)	225	41.8 (37.6–46.1)	
*DGAT1* p.K232A	KK (K)	13	4.8 (2.5–8.1)	130	24.2 (20.6–28.0)	
	KA	104	38.7 (32.8–44.8)			0.411
	AA (A)	152	56.5 (50.4–62.5)	408	75.8 (72.0–79.4)	

CI: 95% confidence interval estimated with the Clopper–Pearson exact method. HWE *p*-values from exact tests.

**Table 3:** Genotype cross-tabulation and LD test results.

		*GC-NPFFR2* rs109452259			LD test	
		CC	CA	AA	r^2^	D′
*GC-NPFFR2*	AA	19	24	5		
rs137147462	AG	27	81	22	0.148	0.425
	GG	11	33	47		
		*BRCA1* rs134817801			LD test	
		AA	AC	CC	r^2^	D′
*GC-NPFFR2*	AA	21	19	8		
rs137147462	AG	40	65	25	0.001	0.044
	GG	31	45	15		
		*DGAT1* p.K232A rs109234250			LD test	
		KK	KA	AA	r^2^	D′
*GC-NPFFR2*	AA	2	16	30		
rs137147462	AG	8	48	74	0.002	0.096
	GG	3	40	48		
		*BRCA1* rs134817801			LD test	
		AA	AC	CC	r^2^	D′
*GC-NPFFR2*	CC	23	30	4		
rs109452259	CA	48	60	30	0.011	0.129
	AA	21	39	14		
		*DGAT1* p.K232A rs109234250			LD test	
		KK	KA	AA	r^2^	D′
*GC-NPFFR2*	CC	2	15	40		
rs109452259	CA	10	51	77	0.013	0.218
	AA	1	38	35		
		*DGAT1* p.K232A rs109234250			LD test	
		KK	KA	AA	r^2^	D′
*BRCA1*	AA	4	41	47		
rs134817801	AC	7	38	84	0.000085	0.014
	CC	2	25	21		

**Table 4:** Summary of inheritance-model effects on SCS.

SNP	Inheritance Model	*p* Value	Genotype	LSM	SE
*GC-NPFFR2* rs137147462	Dominant	0.037	AA	2.32	0.14
*GC-NPFFR2* rs137147462	Dominant	0.037	AG+GG	2.63	0.06
*GC-NPFFR2* rs137147462	Recessive	0.0054	AA+AG	2.46	0.07
*GC-NPFFR2* rs137147462	Recessive	0.0054	GG	2.80	0.10
*DGAT1* p.K232A	Recessive	0.044	KK+KA	2.45	0.09
*DGAT1* p.K232A	Recessive	0.044	AA	2.68	0.08

**Table 5:** Association of *GC-NPFFR2* rs137147462 with mastitis severity.

Genotype	OR	95%CI (Lower-Upper)	*p* Value	Significance
AG	1.35	0.99–1.84	0.0599	ns
GG	1.63	1.18–2.28	0.00354	**

Odds ratios (OR) and 95% confidence intervals (CI) from the ordinal logistic regression model assessing the association between GC-NPFFR2 rs137147462 genotypes and mastitis severity classified into three SCC-based categories (healthy, SCM, and CM). AA genotype was used as the reference category. Significance codes: ** *p* < 0.01; ns = not significant.

## Data Availability

The datasets analyzed during the current study are publicly available at Figshare: https://doi.org/10.6084/m9.figshare.30071734.

## Supplementary Materials

The following supporting information can be downloaded at https://www.mdpi.com/article/10.3390/ani15223239/s1: Table S1: Descriptive statistics; Table S2: Summary of genotype main effects on test-day traits; Table S3: LSM ± SE, Tukey-adjusted groupings, and *p*-values of genotype effects by DIM stage for five milk traits; Table S4: Full results of inheritance-model comparisons for all SNP-trait combinations.
