# Supplementary material for: Recessive Effect of GC-NPFFR2 rs137147462 on Somatic Cell Score (Mastitis Susceptibility) in Japanese Holsteins
Source: Animals (Basel). 2025 Nov 8;15(22):3239. doi: 10.3390/ani15223239 (PMC12649643; doi:10.3390/ani15223239)
Supplement: Supplementary file 1 [file animals-15-03239-s001.zip › animals-3954249-supplementary.pdf]

Supplementary Materials

Supplementary Tables

Table S1. Descriptive statistics

Group	Subgroup	Sample size	Variable	Mean ± SD	Min	Max
Overall	-	N=10729	DIM	184.69±114.76	7	717
			Parity	2.75±1.63	1	10
			Test day Milk Yield (kg)	32.37±9.11	1.8	62.9
			Test day Fat (%)	3.86±0.65	1.68	7.85
			Test day Protein (%)	3.39±0.34	1.3	5.22
			Test day SNF (%)	8.87±0.37	3.67	10.64
			Test day SCS	2.49±1.97	-2.06	10.3
Parity	P1	n=2867	DIM	186.14±115.48	7	717
			Test day Milk Yield (kg)	28.09±5.73	6.4	50.6
			Test day Fat (%)	3.91±0.65	1.68	7.85
			Test day Protein (%)	3.4±0.32	2.55	4.79
			Test day SNF (%)	8.98±0.33	7.46	10.64
			Test day SCS	2.19±1.76	-1.32	10.19
	P2	n=2652	DIM	190.6±117.87	7	710
			Test day Milk Yield (kg)	33.35±9.22	6.1	61.3
			Test day Fat (%)	3.86±0.63	1.82	7.45
			Test day Protein (%)	3.43±0.35	1.3	4.95
			Test day SNF (%)	8.92±0.37	3.67	10.25
			Test day SCS	2.14±1.75	-1.64	9.45
	P3	n=2229	DIM	188.22±115.15	7	687
			Test day Milk Yield (kg)	34.78±10	2.9	62.9
			Test day Fat (%)	3.84±0.65	1.77	7.52
			Test day Protein (%)	3.4±0.36	2.45	5.22
			Test day SNF (%)	8.86±0.37	7.55	10.15
			Test day SCS	2.61±2.05	-2.06	9.68
	P4+	n=2981	DIM	175.4±110.4	7	626
			Test day Milk Yield (kg)	33.83±9.58	1.8	61.1
			Test day Fat (%)	3.82±0.65	1.93	7.64
			Test day Protein (%)	3.32±0.34	2.2	4.85

			Test day SNF (%)	8.75±0.37	6.85	10.14
			Test day SCS	2.99±2.16	-2.06	10.3
DIM	≤50	n=1382	Parity	2.84±1.7	1	10
			Test day Milk Yield (kg)	37.76±8.92	7.4	61.3
			Test day Fat (%)	3.83±0.72	1.93	7.71
			Test day Protein (%)	3.16±0.31	2.2	4.39
			Test day SNF (%)	8.67±0.34	7.6	9.9
			Test day SCS	2.07±1.93	-2.06	10.28
	51-100	n=1624	Parity	2.87±1.74	1	10
			Test day Milk Yield (kg)	38.72±8.54	5.1	62.9
			Test day Fat (%)	3.53±0.57	1.7	5.9
			Test day Protein (%)	3.13±0.26	1.3	4.04
			Test day SNF (%)	8.7±0.35	3.67	9.65
			Test day SCS	2.07±2.09	-2.06	10.3
	101-150	n=1594	Parity	2.75±1.66	1	10
			Test day Milk Yield (kg)	35.92±7.82	7.4	57.3
			Test day Fat (%)	3.68±0.59	1.68	5.53
			Test day Protein (%)	3.31±0.27	2.46	4.2
			Test day SNF (%)	8.85±0.33	7.59	9.81
			Test day SCS	2.18±2.08	-1.64	9.34
	151-200	n=1569	Parity	2.78±1.65	1	10
			Test day Milk Yield (kg)	33.21±7.28	6.4	54.6
			Test day Fat (%)	3.83±0.59	1.96	7.7
			Test day Protein (%)	3.39±0.27	2.49	4.39
			Test day SNF (%)	8.89±0.31	7.83	9.92
			Test day SCS	2.42±1.96	-1.32	9.33
	201-250	n=1499	Parity	2.69±1.62	1	10
			Test day Milk Yield (kg)	29.9±7.01	6.6	54.2
			Test day Fat (%)	3.92±0.59	2.12	7.85
			Test day Protein (%)	3.46±0.28	2.62	5.22
			Test day SNF (%)	8.92±0.32	7.75	10.09
			Test day SCS	2.62±1.87	-1.06	10.02
	251-300	n=1344	Parity	2.65±1.53	1	9
			Test day Milk Yield (kg)	27.35±6.38	5.5	53.5
			Test day Fat (%)	4.03±0.59	2.36	7.45
			Test day Protein (%)	3.54±0.29	2.72	4.59
			Test day SNF (%)	8.98±0.35	7.46	10.15

			Test day SCS	2.8±1.78	-1.32	10.12
	301-350	n=844	Parity	2.69±1.54	1	9
			Test day Milk Yield (kg)	25.05±6.22	7.7	46.4
			Test day Fat (%)	4.14±0.62	2.13	6.52
			Test day Protein (%)	3.64±0.32	2.7	4.77
			Test day SNF (%)	9.05±0.38	7.56	10.18
			Test day SCS	3.06±1.75	-0.64	9.47
	351+	n=873	Parity	2.58±1.43	1	9
			Test day Milk Yield (kg)	23.15±6.01	1.8	42.5
			Test day Fat (%)	4.22±0.63	2.24	7.11
			Test day Protein (%)	3.73±0.32	2.88	4.95
			Test day SNF (%)	9.12±0.4	6.85	10.64
			Test day SCS	3.35±1.72	-0.64	10.29
Season	Spring (Mar–May)	n=2511	DIM	192.48±114.21	7	702
			Parity	2.74±1.63	1	10
			Test day Milk Yield (kg)	32.08±9.06	6.1	61.6
			Test day Fat (%)	3.87±0.64	1.92	7.45
			Test day Protein (%)	3.39±0.33	2.49	4.71
			Test day SNF (%)	8.89±0.36	7.64	10.14
			Test day SCS	2.47±1.91	-1.64	9.47
	Summer (Jun–Aug)	n=2727	DIM	187.91±119.62	7	717
			Parity	2.73±1.59	1	10
			Test day Milk Yield (kg)	33.28±9.37	5.5	62.9
			Test day Fat (%)	3.75±0.63	1.68	7.12
			Test day Protein (%)	3.3±0.33	2.2	4.72
			Test day SNF (%)	8.79±0.36	7.28	10.21
			Test day SCS	2.49±2.04	-2.06	10.29
	Autumn (Sep-Nov)	n=2736	DIM	179.07±114.08	7	687
			Parity	2.76±1.65	1	10
			Test day Milk Yield (kg)	32.05±9.1	1.8	59.6
			Test day Fat (%)	3.83±0.67	1.7	7.71
			Test day Protein (%)	3.41±0.35	2.45	4.77
			Test day SNF (%)	8.89±0.37	6.85	10.16
			Test day SCS	2.52±1.96	-1.64	10.28
	Winter (Dec-Feb)	n=2755	DIM	179.97±110.5	7	677
			Parity	2.74±1.66	1	10
			Test day Milk Yield (kg)	32.07±8.84	6.6	60.4

			Test day Fat (%)	3.98±0.61	1.77	7.85
			Test day Protein (%)	3.44±0.34	1.3	5.22
			Test day SNF (%)	8.93±0.37	3.67	10.64
			Test day SCS	2.47±1.96	-1.64	10.3
Calving Year	2010	n=20	DIM	163.55±93.18	20	336
			Parity	1±0	1	1
			Test day Milk Yield (kg)	21.2±3.54	11.2	25.7
			Test day Fat (%)	4.36±0.46	3.3	5.2
			Test day Protein (%)	3.68±0.17	3.4	3.9
			Test day SNF (%)	9.47±0.11	9.2	9.6
			Test day SCS	1.51±0.42	0.75	2.14
	2011	n=36	DIM	146.94±84.91	9	326
			Parity	1.44±0.5	1	2
			Test day Milk Yield (kg)	23.27±6.23	8.7	37.6
			Test day Fat (%)	4.07±0.31	3.4	4.7
			Test day Protein (%)	3.52±0.19	3.2	3.9
			Test day SNF (%)	9.12±0.18	8.8	9.5
			Test day SCS	2.43±0.89	0.6	4.99
	2012	n=73	DIM	143.34±84.82	7	330
			Parity	1.68±0.81	1	3
			Test day Milk Yield (kg)	24.2±6.51	7.4	40
			Test day Fat (%)	4.01±0.44	2.72	5.04
			Test day Protein (%)	3.46±0.18	2.97	3.88
			Test day SNF (%)	9.05±0.18	8.46	9.41
			Test day SCS	1.86±1.34	-0.18	7.46
	2013	n=146	DIM	160.03±91.75	8	345
			Parity	1.97±1.08	1	4
			Test day Milk Yield (kg)	29.13±6.61	8.8	43.5
			Test day Fat (%)	3.87±0.51	2.68	5.84
			Test day Protein (%)	3.31±0.26	2.71	3.92
			Test day SNF (%)	8.89±0.24	8.18	9.53
			Test day SCS	1.55±1.11	-0.47	5.6
	2014	n=229	DIM	184.19±114.26	9	531

		Parity	2.24±1.1	1	5
		Test day Milk Yield (kg)	30.37±8.94	8.5	53.2
		Test day Fat (%)	3.81±0.6	1.94	5.92
		Test day Protein (%)	3.36±0.26	2.85	4.07
		Test day SNF (%)	8.9±0.26	8.01	9.66
		Test day SCS	2.21±1.57	-1.32	8.6
2015	n=469	DIM	161.7±92.48	7	441
		Parity	1.97±1.35	1	6
		Test day Milk Yield (kg)	29.01±7.52	7.4	54
		Test day Fat (%)	3.84±0.65	2.02	7.64
		Test day Protein (%)	3.37±0.32	2.59	4.61
		Test day SNF (%)	8.93±0.31	8.09	10.11
		Test day SCS	2.5±1.64	-0.47	10.19
2016	n=977	DIM	186.63±111.05	8	549
		Parity	2.03±1.36	1	7
		Test day Milk Yield (kg)	31.22±8.14	3.2	56
		Test day Fat (%)	3.87±0.61	1.99	6.56
		Test day Protein (%)	3.43±0.34	2.6	4.95
		Test day SNF (%)	8.94±0.34	7.55	10.24
		Test day SCS	2.47±1.73	-1.06	9.18
2017	n=1332	DIM	183.39±108.38	7	572
		Parity	2.32±1.53	1	8
		Test day Milk Yield (kg)	31.56±8.98	6.1	60.4
		Test day Fat (%)	3.8±0.62	1.86	7.52
		Test day Protein (%)	3.41±0.33	2.54	5.22
		Test day SNF (%)	8.9±0.35	7.75	10.15
		Test day SCS	2.47±1.89	-2.06	9.68
2018	n=1842	DIM	198.45±122.05	7	717
		Parity	2.58±1.55	1	8
		Test day Milk Yield (kg)	31.8±8.88	1.8	59.9
		Test day Fat (%)	3.87±0.64	1.77	6.86
		Test day Protein (%)	3.42±0.32	1.3	4.72

		Test day SNF (%)	8.9±0.38	3.67	10.19
		Test day SCS	2.42±2.02	-1.64	10.29
2019	n=2254	DIM	192.3±123.25	7	687
		Parity	2.55±1.74	1	9
		Test day Milk Yield (kg)	32.17±9.01	2.9	60.4
		Test day Fat (%)	3.97±0.67	1.68	7.85
		Test day Protein (%)	3.32±0.34	2.2	4.85
		Test day SNF (%)	8.8±0.36	7.46	10.64
		Test day SCS	2.48±2.04	-1.64	10.02
2020	n=1568	DIM	183.22±109.1	7	622
		Parity	3.23±1.68	1	10
		Test day Milk Yield (kg)	34.17±9.81	5.5	62.9
		Test day Fat (%)	3.81±0.63	1.96	7.45
		Test day Protein (%)	3.36±0.36	2.38	4.77
		Test day SNF (%)	8.83±0.4	7.52	10.16
		Test day SCS	2.56±2.09	-1.64	10.3
2021	n=934	DIM	178.87±116.05	7	702
		Parity	3.4±1.11	2	8
		Test day Milk Yield (kg)	34.19±9.1	5.1	61
		Test day Fat (%)	3.79±0.65	1.81	5.83
		Test day Protein (%)	3.44±0.38	2.46	4.77
		Test day SNF (%)	8.92±0.42	7.57	10.25
		Test day SCS	2.65±2.09	-1.64	8.97
2022	n=506	DIM	183.57±117.75	7	626
		Parity	4.03±1	3	7
		Test day Milk Yield (kg)	35.36±9.07	9.1	61.6
		Test day Fat (%)	3.82±0.67	2.06	7.2
		Test day Protein (%)	3.41±0.38	2.49	4.54
		Test day SNF (%)	8.89±0.4	7.84	10.14
		Test day SCS	2.86±2.12	-2.06	10.28
2023	n=269	DIM	150.97±94.53	9	380
		Parity	4.74±0.93	4	8

		Test day Milk Yield (kg)	35.89±8.94	15.1	58.5
		Test day Fat (%)	3.59±0.68	1.93	5.68
		Test day Protein (%)	3.34±0.36	2.49	4.31
		Test day SNF (%)	8.86±0.36	7.73	10.05
		Test day SCS	2.48±2.11	-1.64	10.12
2024	n=71	DIM	104.86±74.39	8	303
		Parity	5.3±0.7	4	6
		Test day Milk Yield (kg)	38.12±7.53	23.3	56.8
		Test day Fat (%)	3.68±0.69	2.04	5.58
		Test day Protein (%)	3.27±0.33	2.77	4.35
		Test day SNF (%)	8.83±0.36	8.01	9.91
		Test day SCS	2.24±1.57	-0.64	6.1
2025	n=3	DIM	65.33±33.01	32	98
		Parity	6±0	6	6
		Test day Milk Yield (kg)	37.13±3.01	33.7	39.3
		Test day Fat (%)	4.03±0.46	3.63	4.53
		Test day Protein (%)	3.35±0.04	3.32	3.39
		Test day SNF (%)	8.92±0.11	8.81	9.02
		Test day SCS	5.06±2.65	2.33	7.62

DIM stages: 5–50, 51–100, 101–150, 151–200, 201–250, 251–300, 301–350, >350 days.

Parity: first, second, third, and ≥fourth.

Season: spring (Mar–May), summer (Jun–Aug), autumn (Sep–Nov), winter (Dec–Feb).

Table S2. Summary of genotype main effects on test-day traits

Variable	SNP	Genotype	LSM	SE	LCL	UCL	Tukey Group	*p*-value
SCS	*GC-NPFFR2* rs137147462	AA	2.31	0.14	2.04	2.58	a	
SCS	*GC-NPFFR2* rs137147462	AG	2.51	0.08	2.35	2.67	ab	0.00999767
SCS	*GC-NPFFR2* rs137147462	GG	2.80	0.10	2.61	2.99	b	

SCS	*GC-NPFFR2* rs109452259	AA	2.65	0.11	2.43	2.86	-	
SCS	*GC-NPFFR2* rs109452259	AC	2.54	0.08	2.38	2.70	-	0.71535998
SCS	*GC-NPFFR2* rs109452259	CC	2.58	0.13	2.33	2.83	-	
SCS	*BRCA1* rs134817801	AA	2.46	0.10	2.27	2.66	-	
SCS	*BRCA1* rs134817801	AC	2.68	0.08	2.51	2.84	-	0.24220971
SCS	*BRCA1* rs134817801	CC	2.53	0.14	2.26	2.79	-	
SCS	*DGAT1* p.K232A	AA	2.68	0.08	2.53	2.83	-	
SCS	*DGAT1* p.K232A	KA	2.46	0.09	2.28	2.64	-	0.11642264
SCS	*DGAT1* p.K232A	KK	2.31	0.26	1.80	2.83	-	
Milk Yield (kg)	*GC-NPFFR2* rs137147462	AA	31.62	0.55	30.54	32.70	-	
Milk Yield (kg)	*GC-NPFFR2* rs137147462	AG	31.56	0.33	30.91	32.21	-	0.96097593
Milk Yield (kg)	*GC-NPFFR2* rs137147462	GG	31.70	0.39	30.93	32.47	-	
Milk Yield (kg)	*GC-NPFFR2* rs109452259	AA	32.16	0.43	31.32	33.01	-	
Milk Yield (kg)	*GC-NPFFR2* rs109452259	AC	31.63	0.32	31.00	32.25	-	0.1379405
Milk Yield (kg)	*GC-NPFFR2* rs109452259	CC	30.85	0.50	29.87	31.83	-	
Milk Yield (kg)	*BRCA1* rs134817801	AA	31.94	0.40	31.16	32.72	-	
Milk Yield (kg)	*BRCA1* rs134817801	AC	31.64	0.33	30.99	32.29	-	0.3426593
Milk Yield (kg)	*BRCA1* rs134817801	CC	30.96	0.54	29.90	32.02	-	
Milk Yield (kg)	*DGAT1* p.K232A	AA	32.26	0.29	31.69	32.84	a	7.4466E-05

Milk Yield (kg)	*DGAT1* p.K232A	KA	31.08	0.36	30.38	31.78	B	
Milk Yield (kg)	*DGAT1* p.K232A	KK	28.05	1.01	26.08	30.02	c	
Fat %	*GC-NPFFR2* rs137147462	AA	3.91	0.05	3.81	4.01	-	
Fat %	*GC-NPFFR2* rs137147462	AG	3.92	0.03	3.86	3.98	-	0.9630358
Fat %	*GC-NPFFR2* rs137147462	GG	3.90	0.04	3.83	3.98	-	
Fat %	*GC-NPFFR2* rs109452259	AA	3.89	0.04	3.81	3.97	-	
Fat %	*GC-NPFFR2* rs109452259	AC	3.93	0.03	3.87	3.98	-	0.7928707
Fat %	*GC-NPFFR2* rs109452259	CC	3.91	0.05	3.81	4.00	-	
Fat %	*BRCA1* rs134817801	AA	3.93	0.04	3.86	4.00	ab	
Fat %	*BRCA1* rs134817801	AC	3.86	0.03	3.80	3.92	A	0.02962095
Fat %	*BRCA1* rs134817801	CC	4.01	0.05	3.92	4.11	b	
Fat %	*DGAT1* p.K232A	AA	3.74	0.02	3.69	3.78	a	
Fat %	*DGAT1* p.K232A	KA	4.10	0.03	4.04	4.15	b	9.0382E-31
Fat %	*DGAT1* p.K232A	KK	4.51	0.07	4.36	4.65	c	
Protein %	*GC-NPFFR2* rs137147462	AA	3.48	0.03	3.42	3.54	-	
Protein %	*GC-NPFFR2* rs137147462	AG	3.44	0.02	3.40	3.48	-	0.17195797
Protein %	*GC-NPFFR2* rs137147462	GG	3.41	0.02	3.37	3.45	-	
Protein %	*GC-NPFFR2* rs109452259	AA	3.41	0.02	3.36	3.46	-	
Protein %	*GC-NPFFR2* rs109452259	AC	3.45	0.02	3.41	3.48	-	0.43138358

Protein %	*GC-NPFFR2* rs109452259	CC	3.43	0.03	3.38	3.49	-	
Protein %	*BRCA1* rs134817801	AA	3.46	0.02	3.42	3.50	-	
Protein %	*BRCA1* rs134817801	AC	3.41	0.02	3.37	3.44	-	0.07257061
Protein %	*BRCA1* rs134817801	CC	3.47	0.03	3.41	3.53	-	
Protein %	*DGAT1* p.K232A	AA	3.36	0.02	3.33	3.39	a	
Protein %	*DGAT1* p.K232A	KA	3.52	0.02	3.49	3.56	a	4.3004E-13
Protein %	*DGAT1* p.K232A	KK	3.63	0.05	3.53	3.73	b	
SNF %	*GC-NPFFR2* rs137147462	AA	8.96	0.04	8.88	9.03	-	
SNF %	*GC-NPFFR2* rs137147462	AG	8.93	0.02	8.89	8.98	-	0.04729475
SNF %	*GC-NPFFR2* rs137147462	GG	8.86	0.03	8.81	8.91	-	
SNF %	*GC-NPFFR2* rs109452259	AA	8.86	0.03	8.80	8.92	-	
SNF %	*GC-NPFFR2* rs109452259	AC	8.94	0.02	8.90	8.98	-	0.09101351
SNF %	*GC-NPFFR2* rs109452259	CC	8.91	0.03	8.84	8.97	-	
SNF %	*BRCA1* rs134817801	AA	8.94	0.03	8.89	9.00	-	
SNF %	*BRCA1* rs134817801	AC	8.88	0.02	8.83	8.92	-	0.07928633
SNF %	*BRCA1* rs134817801	CC	8.95	0.04	8.88	9.02	-	
SNF %	*DGAT1* p.K232A	AA	8.82	0.02	8.78	8.86	a	
SNF %	*DGAT1* p.K232A	KA	9.02	0.02	8.97	9.06	a	9.9033E-12
SNF %	*DGAT1* p.K232A	KK	9.14	0.07	9.01	9.26	b	

Least squares means (LSMs), standard errors (SE), 95% confidence intervals (LCL–UCL), Tukey's HSD groupings, and ANOVA *p*-values for genotype main effects are shown for each candidate SNP and test-day variable.
A different letter in the Tukey Group column indicates a significant difference at P < 0.05.

Table S3. LSM ± SE, Tukey-adjusted groupings, and p-values of genotype effects by DIM stage for five milk traits.

Variable	SNP	DIM stage(days)	Genotype	Genotype class	n	LSM	SE	Lower CI	Upper CI	Tukey group	$P_{DIM\ stage}$	$P_{genotype}$	$P_{ParityGroup}$	P_{Season}	$P_{CalvingYear}$	$P_{(DIM \times Genotype\ interaction)}$
SCS	*GC-NPFFR2* rs137147462	0-50	AA	Wild	248	1.684263	0.170058	1.350954	2.017571	-						
SCS	*GC-NPFFR2* rs137147462	0-50	AG	Heterozygote	687	2.111269	0.102441	1.910488	2.31205	-						
SCS	*GC-NPFFR2* rs137147462	0-50	GG	Mutant	447	2.170496	0.123586	1.928272	2.41272	-						
SCS	*GC-NPFFR2* rs137147462	51-100	AA	Wild	289	1.784985	0.165477	1.460656	2.109314	-						
SCS	*GC-NPFFR2* rs137147462	51-100	AG	Heterozygote	789	2.039314	0.099596	1.844109	2.234518	-						
SCS	*GC-NPFFR2* rs137147462	51-100	GG	Mutant	546	2.161377	0.118614	1.928899	2.393856	-						
SCS	*GC-NPFFR2* rs137147462	101-150	AA	Wild	284	1.966284	0.165664	1.641589	2.290979	-	2.83E-106	0.00945161	7.39E-22	0.474588553	2.39E-08	0.406543865
SCS	*GC-NPFFR2* rs137147462	101-150	AG	Heterozygote	797	2.084067	0.099466	1.889116	2.279017	-						
SCS	*GC-NPFFR2* rs137147462	101-150	GG	Mutant	513	2.427453	0.120065	2.192129	2.662777	-						
SCS	*GC-NPFFR2* rs137147462	151-200	AA	Wild	278	2.218483	0.166648	1.891858	2.545108	ab						
SCS	*GC-NPFFR2* rs137147462	151-200	AG	Heterozygote	752	2.269875	0.100481	2.072936	2.466813	a						
SCS	*GC-NPFFR2* rs137147462	151-200	GG	Mutant	539	2.688696	0.11895	2.455559	2.921833	b						
SCS	*GC-NPFFR2* rs137147462	201-250	AA	Wild	261	2.344249	0.168225	2.014534	2.673964	a						

SCS	*GC-NPFFR2* rs137147462	201-250	AG	Heterozygote	734	2.513806	0.100939	2.31597	2.711643	a						
SCS	*GC-NPFFR2* rs137147462	201-250	GG	Mutant	504	2.913718	0.120546	2.677452	3.149983	b						
SCS	*GC-NPFFR2* rs137147462	251-300	AA	Wild	238	2.549348	0.171725	2.212774	2.885922	a						
SCS	*GC-NPFFR2* rs137147462	251-300	AG	Heterozygote	660	2.803703	0.102915	2.601994	3.005413	ab						
SCS	*GC-NPFFR2* rs137147462	251-300	GG	Mutant	446	3.06202	0.123618	2.819734	3.304306	b						
SCS	*GC-NPFFR2* rs137147462	301-350	AA	Wild	137	2.859276	0.196366	2.474405	3.244146	-						
SCS	*GC-NPFFR2* rs137147462	301-350	AG	Heterozygote	414	2.986779	0.115022	2.76134	3.212218	-						
SCS	*GC-NPFFR2* rs137147462	301-350	GG	Mutant	293	3.350974	0.136795	3.082861	3.619087	-						
SCS	*GC-NPFFR2* rs137147462	351+	AA	Wild	118	3.098767	0.208007	2.69108	3.506453	-						
SCS	*GC-NPFFR2* rs137147462	351+	AG	Heterozygote	447	3.285652	0.114748	3.06075	3.510554	-						
SCS	*GC-NPFFR2* rs137147462	351+	GG	Mutant	308	3.63076	0.136906	3.362429	3.899091	-						
SCS	*GC-NPFFR2* rs109452259	0-50	CC	Wild	302	1.906199	0.15674	1.598994	2.213404	-						
SCS	*GC-NPFFR2* rs109452259	0-50	CA	Heterozygote	706	2.017116	0.101148	1.818869	2.215363	-						
SCS	*GC-NPFFR2* rs109452259	0-50	AA	Mutant	374	2.263122	0.137536	1.993558	2.532687	-						
SCS	*GC-NPFFR2* rs109452259	51-100	CC	Wild	317	2.058732	0.155468	1.754021	2.363443	-	2.13E-104	0.74195802	1.89E-22	0.488542758	9.22E-08	0.092201946
SCS	*GC-NPFFR2* rs109452259	51-100	CA	Heterozygote	862	2.039845	0.097353	1.849035	2.230654	-						
SCS	*GC-NPFFR2* rs109452259	51-100	AA	Mutant	445	2.014208	0.132856	1.753814	2.274601	-						
SCS	*GC-NPFFR2* rs109452259	101-150	CC	Wild	342	2.232416	0.153101	1.932343	2.532488	-						

SCS	*GC-NPFFR2*<br>rs109452259	101-150	CA	Heterozygote	81<br>1	2.10546<br>3	0.09839<br>8	1.912606	2.29832	-						
SCS	*GC-NPFFR2*<br>rs109452259	101-150	AA	Mutant	44<br>1	2.27739<br>7	0.13320<br>1	2.016329	2.538466	-						
SCS	*GC-NPFFR2*<br>rs109452259	151-200	CC	Wild	32<br>2	2.58675<br>6	0.15472	2.283511	2.890002	-						
SCS	*GC-NPFFR2*<br>rs109452259	151-200	CA	Heterozygote	80<br>5	2.30168<br>2	0.09857<br>5	2.10848	2.494885	-						
SCS	*GC-NPFFR2*<br>rs109452259	151-200	AA	Mutant	44<br>2	2.46260<br>3	0.13309<br>3	2.201745	2.723461	-						
SCS	*GC-NPFFR2*<br>rs109452259	201-250	CC	Wild	31<br>5	2.74332<br>1	0.15523<br>3	2.439069	3.047573	-						
SCS	*GC-NPFFR2*<br>rs109452259	201-250	CA	Heterozygote	77<br>2	2.54890<br>3	0.09934<br>2	2.354196	2.743609	-						
SCS	*GC-NPFFR2*<br>rs109452259	201-250	AA	Mutant	41<br>2	2.65850<br>3	0.13486<br>4	2.394174	2.922832	-						
SCS	*GC-NPFFR2*<br>rs109452259	251-300	CC	Wild	27<br>3	2.83660<br>2	0.16003<br>8	2.522935	3.15027	-						
SCS	*GC-NPFFR2*<br>rs109452259	251-300	CA	Heterozygote	69<br>6	2.78306<br>5	0.10115<br>5	2.584805	2.981325	-						
SCS	*GC-NPFFR2*<br>rs109452259	251-300	AA	Mutant	37<br>5	2.97501<br>7	0.13746<br>6	2.705589	3.244444	-						
SCS	*GC-NPFFR2*<br>rs109452259	301-350	CC	Wild	15<br>0	2.85766<br>7	0.18536<br>4	2.49436	3.220973	-						
SCS	*GC-NPFFR2*<br>rs109452259	301-350	CA	Heterozygote	42<br>2	3.12542<br>4	0.11393	2.902125	3.348723	-						
SCS	*GC-NPFFR2*<br>rs109452259	301-350	AA	Mutant	27<br>2	3.16772<br>6	0.14764<br>7	2.878343	3.457109	-						
SCS	*GC-NPFFR2*<br>rs109452259	351+	CC	Wild	15<br>3	3.22277<br>2	0.1873	2.855671	3.589874	-						
SCS	*GC-NPFFR2*<br>rs109452259	351+	CA	Heterozygote	44<br>5	3.43639<br>5	0.11451<br>3	3.211955	3.660836	-						
SCS	*GC-NPFFR2*<br>rs109452259	351+	AA	Mutant	27<br>5	3.36340<br>6	0.14909<br>6	3.071183	3.65563	-						
SCS	*BRCA1* rs134817801	0-50	AA	Wild	44<br>3	2.04980<br>2	0.12585<br>8	1.803125	2.296479	-	6.48E-<br>117	0.1993152<br>8	3.55E-22	0.31946791<br>5	7.39E-08	0.002653709

SCS	*BRCA1* rs134817801	0-50	AC	Heterozygote	670	2.120414	0.103643	1.917277	2.323551	-
SCS	*BRCA1* rs134817801	0-50	CC	Mutant	269	1.93714	0.166758	1.610301	2.263979	-
SCS	*BRCA1* rs134817801	51-100	AA	Wild	527	2.049456	0.121608	1.811108	2.287805	-
SCS	*BRCA1* rs134817801	51-100	AC	Heterozygote	788	2.064975	0.100409	1.868176	2.261774	-
SCS	*BRCA1* rs134817801	51-100	CC	Mutant	309	1.963016	0.162453	1.644614	2.281418	-
SCS	*BRCA1* rs134817801	101-150	AA	Wild	512	2.142023	0.12226	1.902398	2.381648	-
SCS	*BRCA1* rs134817801	101-150	AC	Heterozygote	768	2.289865	0.100854	2.092194	2.487535	-
SCS	*BRCA1* rs134817801	101-150	CC	Mutant	314	1.982823	0.162074	1.665164	2.300482	-
SCS	*BRCA1* rs134817801	151-200	AA	Wild	491	2.329513	0.123075	2.08829	2.570736	-
SCS	*BRCA1* rs134817801	151-200	AC	Heterozygote	760	2.530689	0.101117	2.332504	2.728875	-
SCS	*BRCA1* rs134817801	151-200	CC	Mutant	318	2.239204	0.161668	1.922341	2.556067	-
SCS	*BRCA1* rs134817801	201-250	AA	Wild	490	2.491787	0.123023	2.250667	2.732907	-
SCS	*BRCA1* rs134817801	201-250	AC	Heterozygote	702	2.717853	0.102643	2.516676	2.91903	-
SCS	*BRCA1* rs134817801	201-250	CC	Mutant	307	2.605251	0.162679	2.286405	2.924096	-
SCS	*BRCA1* rs134817801	251-300	AA	Wild	432	2.637536	0.126049	2.390486	2.884587	-
SCS	*BRCA1* rs134817801	251-300	AC	Heterozygote	649	2.988213	0.104214	2.783958	3.192468	-
SCS	*BRCA1* rs134817801	251-300	CC	Mutant	263	2.860968	0.167557	2.532561	3.189374	-
SCS	*BRCA1* rs134817801	301-350	AA	Wild	271	2.79672	0.141062	2.520244	3.073197	a

SCS	*BRCA1* rs134817801	301-350	AC	Heterozygote	427	3.279429	0.114943	3.054146	3.504712	b						
SCS	*BRCA1* rs134817801	301-350	CC	Mutant	146	3.106038	0.192378	2.728984	3.483092	ab						
SCS	*BRCA1* rs134817801	351+	AA	Wild	314	3.068848	0.138939	2.796534	3.341163	a						
SCS	*BRCA1* rs134817801	351+	AC	Heterozygote	422	3.439721	0.116565	3.211258	3.668183	ab						
SCS	*BRCA1* rs134817801	351+	CC	Mutant	137	3.833759	0.198803	3.444112	4.223406	b						
SCS	*DGAT1* p.K232A	0-50	KK	Wild	68	1.774175	0.325675	1.135863	2.412487	-						
SCS	*DGAT1* p.K232A	0-50	KA	Heterozygote	538	1.971888	0.115638	1.745242	2.198534	-						
SCS	*DGAT1* p.K232A	0-50	AA	Mutant	776	2.143479	0.095786	1.955741	2.331216	-						
SCS	*DGAT1* p.K232A	51-100	KK	Wild	68	2.21121	0.32509	1.574046	2.848374	-						
SCS	*DGAT1* p.K232A	51-100	KA	Heterozygote	619	1.864694	0.112292	1.644607	2.084782	-						
SCS	*DGAT1* p.K232A	51-100	AA	Mutant	937	2.144662	0.092422	1.963519	2.325804	-						
SCS	*DGAT1* p.K232A	101-150	KK	Wild	65	1.713883	0.328163	1.070695	2.35707	-						
SCS	*DGAT1* p.K232A	101-150	KA	Heterozygote	616	2.034136	0.112572	1.8135	2.254772	-	8.81E-35	0.11040039	2.43E-22	0.502191464	6.34E-08	0.381475767
SCS	*DGAT1* p.K232A	101-150	AA	Mutant	913	2.315507	0.092775	2.133672	2.497341	-						
SCS	*DGAT1* p.K232A	151-200	KK	Wild	71	2.077775	0.321904	1.446854	2.708695	-						
SCS	*DGAT1* p.K232A	151-200	KA	Heterozygote	628	2.278955	0.112071	2.059299	2.498611	-						
SCS	*DGAT1* p.K232A	151-200	AA	Mutant	870	2.519327	0.093657	2.335763	2.702891	-						
SCS	*DGAT1* p.K232A	201-250	KK	Wild	58	2.228247	0.336347	1.56902	2.887474	-						
SCS	*DGAT1* p.K232A	201-250	KA	Heterozygote	582	2.61618	0.113649	2.393431	2.838929	-						

SCS	*DGAT1* p.K232A	201-250	AA	Mutant	859	2.653769	0.09385	2.469827	2.837712	-						
SCS	*DGAT1* p.K232A	251-300	KK	Wild	60	2.422276	0.332792	1.770016	3.074535	-						
SCS	*DGAT1* p.K232A	251-300	KA	Heterozygote	522	2.78036	0.116112	2.552785	3.007935	-						
SCS	*DGAT1* p.K232A	251-300	AA	Mutant	762	2.928528	0.096035	2.740303	3.116753	-						
SCS	*DGAT1* p.K232A	301-350	KK	Wild	34	3.025655	0.386091	2.26893	3.78238	-						
SCS	*DGAT1* p.K232A	301-350	KA	Heterozygote	366	2.956642	0.125951	2.709782	3.203501	-						
SCS	*DGAT1* p.K232A	301-350	AA	Mutant	444	3.189925	0.109372	2.975559	3.40429	-						
SCS	*DGAT1* p.K232A	351+	KK	Wild	27	3.102006	0.436448	2.246584	3.957429	ab						
SCS	*DGAT1* p.K232A	351+	KA	Heterozygote	369	3.157967	0.127713	2.907655	3.408279	a						
SCS	*DGAT1* p.K232A	351+	AA	Mutant	477	3.551179	0.108933	3.337675	3.764683	b						
MilkYield	*GC-NPFFR2* rs137147462	0-50	AA	Wild	248	37.77283	0.657134	36.48487	39.06079	-						
MilkYield	*GC-NPFFR2* rs137147462	0-50	AG	Heterozygote	687	38.11623	0.396038	37.34001	38.89245	-						
MilkYield	*GC-NPFFR2* rs137147462	0-50	GG	Mutant	447	38.59528	0.476918	37.66054	39.53002	-						
MilkYield	*GC-NPFFR2* rs137147462	51-100	AA	Wild	289	38.9177	0.642198	37.65902	40.17639	-	0	0.96673012	8.28E-232	5.59E-30	0.75640321	0.005693041
MilkYield	*GC-NPFFR2* rs137147462	51-100	AG	Heterozygote	789	39.15064	0.38661	38.39289	39.90838	-						
MilkYield	*GC-NPFFR2* rs137147462	51-100	GG	Mutant	546	39.41574	0.46046	38.51325	40.31822	-						
MilkYield	*GC-NPFFR2* rs137147462	101-150	AA	Wild	284	36.77616	0.642743	35.51641	38.03592	-						
MilkYield	*GC-NPFFR2* rs137147462	101-150	AG	Heterozygote	797	36.35171	0.386197	35.59478	37.10864	-						

MilkYield	*GC-NPFFR2* rs137147462	101-150	GG	Mutant	513	37.00357	0.465251	36.09169	37.91544	-						
MilkYield	*GC-NPFFR2* rs137147462	151-200	AA	Wild	278	33.39934	0.646032	32.13314	34.66553	-						
MilkYield	*GC-NPFFR2* rs137147462	151-200	AG	Heterozygote	752	33.73974	0.389526	32.97628	34.5032	-						
MilkYield	*GC-NPFFR2* rs137147462	151-200	GG	Mutant	539	34.05651	0.461571	33.15185	34.96117	-						
MilkYield	*GC-NPFFR2* rs137147462	201-250	AA	Wild	261	30.38586	0.651176	29.10958	31.66214	-						
MilkYield	*GC-NPFFR2* rs137147462	201-250	AG	Heterozygote	734	30.37986	0.391034	29.61345	31.14627	-						
MilkYield	*GC-NPFFR2* rs137147462	201-250	GG	Mutant	504	30.66041	0.466852	29.74539	31.57542	-						
MilkYield	*GC-NPFFR2* rs137147462	251-300	AA	Wild	238	27.89178	0.662807	26.59271	29.19086	-						
MilkYield	*GC-NPFFR2* rs137147462	251-300	AG	Heterozygote	660	27.64802	0.397515	26.8689	28.42713	-						
MilkYield	*GC-NPFFR2* rs137147462	251-300	GG	Mutant	446	27.98676	0.477021	27.05182	28.9217	-						
MilkYield	*GC-NPFFR2* rs137147462	301-350	AA	Wild	137	25.59036	0.745225	24.12975	27.05098	-						
MilkYield	*GC-NPFFR2* rs137147462	301-350	AG	Heterozygote	414	25.05132	0.437932	24.19299	25.90965	-						
MilkYield	*GC-NPFFR2* rs137147462	301-350	GG	Mutant	293	24.63323	0.521004	23.61209	25.65438	-						
MilkYield	*GC-NPFFR2* rs137147462	351+	AA	Wild	118	23.2635	0.784829	21.72526	24.80173	b						
MilkYield	*GC-NPFFR2* rs137147462	351+	AG	Heterozygote	447	22.22839	0.437187	21.37152	23.08526	ab						
MilkYield	*GC-NPFFR2* rs137147462	351+	GG	Mutant	308	20.64768	0.521526	19.62551	21.66985	a						
MilkYield	*GC-NPFFR2* rs109452259	0-50	CC	Wild	302	37.57352	0.595445	36.40647	38.74057	-	0	0.18194811	4.18E-231	1.33E-29	0.66339109	0.541196534
MilkYield	*GC-NPFFR2* rs109452259	0-50	CA	Heterozygote	706	38.11458	0.383948	37.36206	38.86711	-						

MilkYield	*GC-NPFFR2* rs109452259	0-50	AA	Mutant	374	38.85098	0.522082	37.82772	39.87424	-
MilkYield	*GC-NPFFR2* rs109452259	51-100	CC	Wild	317	38.4183	0.591246	37.25948	39.57713	-
MilkYield	*GC-NPFFR2* rs109452259	51-100	CA	Heterozygote	862	39.097	0.371145	38.36957	39.82443	-
MilkYield	*GC-NPFFR2* rs109452259	51-100	AA	Mutant	445	39.96037	0.506284	38.96807	40.95266	-
MilkYield	*GC-NPFFR2* rs109452259	101-150	CC	Wild	342	35.73701	0.583254	34.59385	36.88017	-
MilkYield	*GC-NPFFR2* rs109452259	101-150	CA	Heterozygote	811	36.65084	0.374647	35.91654	37.38513	-
MilkYield	*GC-NPFFR2* rs109452259	101-150	AA	Mutant	441	37.28218	0.50746	36.28758	38.27678	-
MilkYield	*GC-NPFFR2* rs109452259	151-200	CC	Wild	322	32.72837	0.588684	31.57457	33.88217	-
MilkYield	*GC-NPFFR2* rs109452259	151-200	CA	Heterozygote	805	33.90787	0.375248	33.1724	34.64334	-
MilkYield	*GC-NPFFR2* rs109452259	151-200	AA	Mutant	442	34.33602	0.507091	33.34214	35.3299	-
MilkYield	*GC-NPFFR2* rs109452259	201-250	CC	Wild	315	29.81056	0.5904	28.6534	30.96772	-
MilkYield	*GC-NPFFR2* rs109452259	201-250	CA	Heterozygote	772	30.42194	0.377847	29.68137	31.1625	-
MilkYield	*GC-NPFFR2* rs109452259	201-250	AA	Mutant	412	31.06099	0.51305	30.05544	32.06655	-
MilkYield	*GC-NPFFR2* rs109452259	251-300	CC	Wild	273	26.49817	0.606663	25.30913	27.68721	-
MilkYield	*GC-NPFFR2* rs109452259	251-300	CA	Heterozygote	696	28.04805	0.383906	27.2956	28.80049	-
MilkYield	*GC-NPFFR2* rs109452259	251-300	AA	Mutant	375	28.30158	0.521844	27.27878	29.32437	-
MilkYield	*GC-NPFFR2* rs109452259	301-350	CC	Wild	150	24.53497	0.693031	23.17665	25.89328	-
MilkYield	*GC-NPFFR2* rs109452259	301-350	CA	Heterozygote	422	24.88424	0.427364	24.04662	25.72186	-

MilkYield	*GC-NPFFR2* rs109452259	301-350	AA	Mutant	272	25.56236	0.556352	24.47194	26.65279	-						
MilkYield	*GC-NPFFR2* rs109452259	351+	CC	Wild	153	22.01344	0.699861	20.64174	23.38514	-						
MilkYield	*GC-NPFFR2* rs109452259	351+	CA	Heterozygote	445	21.88696	0.42951	21.04514	22.72879	-						
MilkYield	*GC-NPFFR2* rs109452259	351+	AA	Mutant	275	21.7199	0.561404	20.61956	22.82023	-						
MilkYield	*BRCA1* rs134817801	0-50	AA	Wild	443	38.33833	0.479432	37.39866	39.278	-						
MilkYield	*BRCA1* rs134817801	0-50	AC	Heterozygote	670	38.24266	0.395751	37.467	39.01832	-						
MilkYield	*BRCA1* rs134817801	0-50	CC	Mutant	269	37.84573	0.637931	36.59541	39.09605	-						
MilkYield	*BRCA1* rs134817801	51-100	AA	Wild	527	39.14541	0.465163	38.23371	40.05712	-						
MilkYield	*BRCA1* rs134817801	51-100	AC	Heterozygote	788	39.53095	0.384933	38.77649	40.2854	-						
MilkYield	*BRCA1* rs134817801	51-100	CC	Mutant	309	38.353	0.623519	37.13092	39.57507	-						
MilkYield	*BRCA1* rs134817801	101-150	AA	Wild	512	36.47387	0.467337	35.55791	37.38984	-						
											0	0.2564206	2.02E-232	3.51E-31	0.85087651	0.001020483
MilkYield	*BRCA1* rs134817801	101-150	AC	Heterozygote	768	37.01389	0.386418	36.25652	37.77125	-						
MilkYield	*BRCA1* rs134817801	101-150	CC	Mutant	314	35.89526	0.622281	34.67561	37.11491	-						
MilkYield	*BRCA1* rs134817801	151-200	AA	Wild	491	33.82623	0.470044	32.90496	34.7475	-						
MilkYield	*BRCA1* rs134817801	151-200	AC	Heterozygote	760	33.85606	0.387299	33.09697	34.61515	-						
MilkYield	*BRCA1* rs134817801	151-200	CC	Mutant	318	33.43615	0.620917	32.21917	34.65312	-						
MilkYield	*BRCA1* rs134817801	201-250	AA	Wild	490	30.85563	0.469844	29.93475	31.77651	-						
MilkYield	*BRCA1* rs134817801	201-250	AC	Heterozygote	702	30.34207	0.392422	29.57293	31.1112	-						

MilkYield	*BRCA1* rs134817801	201-250	CC	Mutant	307	30.05522	0.624285	28.83164	31.2788	-						
MilkYield	*BRCA1* rs134817801	251-300	AA	Wild	432	28.28622	0.479973	27.34549	29.22695	-						
MilkYield	*BRCA1* rs134817801	251-300	AC	Heterozygote	649	27.76937	0.397663	26.98996	28.54877	-						
MilkYield	*BRCA1* rs134817801	251-300	CC	Mutant	263	26.99013	0.640621	25.73453	28.24572	-						
MilkYield	*BRCA1* rs134817801	301-350	AA	Wild	271	25.68976	0.530782	24.64944	26.73007	-						
MilkYield	*BRCA1* rs134817801	301-350	AC	Heterozygote	427	24.70488	0.433905	23.85444	25.55532	-						
MilkYield	*BRCA1* rs134817801	301-350	CC	Mutant	146	24.55879	0.724505	23.13879	25.9788	-						
MilkYield	*BRCA1* rs134817801	351+	AA	Wild	314	23.43288	0.523791	22.40627	24.45949	b						
MilkYield	*BRCA1* rs134817801	351+	AC	Heterozygote	422	21.26723	0.439507	20.40581	22.12864	a						
MilkYield	*BRCA1* rs134817801	351+	CC	Mutant	137	20.08229	0.746579	18.61903	21.54556	a						
MilkYield	*DGAT1* p.K232A	0-50	KK	Wild	68	34.461	1.21794	32.07388	36.84812	a						
MilkYield	*DGAT1* p.K232A	0-50	KA	Heterozygote	538	37.69683	0.432381	36.84938	38.54429	b						
MilkYield	*DGAT1* p.K232A	0-50	AA	Mutant	776	38.8641	0.358116	38.16221	39.566	b						
MilkYield	*DGAT1* p.K232A	51-100	KK	Wild	68	34.674	1.215894	32.29089	37.05711	a						
											0	2.31E-04	5.14E-230	3.47E-29	0.454164	0.165094107
MilkYield	*DGAT1* p.K232A	51-100	KA	Heterozygote	619	38.5337	0.420893	37.70876	39.35864	b						
MilkYield	*DGAT1* p.K232A	51-100	AA	Mutant	937	40.00027	0.346625	39.3209	40.67964	c						
MilkYield	*DGAT1* p.K232A	101-150	KK	Wild	65	32.89395	1.226436	30.49018	35.29772	a						
MilkYield	*DGAT1* p.K232A	101-150	KA	Heterozygote	616	35.85625	0.421871	35.0294	36.6831	a						

MilkYield	*DGAT1* p.K232A	101-150	AA	Mutant	913	37.46213	0.347816	36.78043	38.14384	b						
MilkYield	*DGAT1* p.K232A	151-200	KK	Wild	71	29.71719	1.204952	27.35553	32.07885	a						
MilkYield	*DGAT1* p.K232A	151-200	KA	Heterozygote	628	33.15945	0.420158	32.33595	33.98295	b						
MilkYield	*DGAT1* p.K232A	151-200	AA	Mutant	870	34.54414	0.350831	33.85652	35.23175	c						
MilkYield	*DGAT1* p.K232A	201-250	KK	Wild	58	26.70935	1.254545	24.25049	29.16822	a						
MilkYield	*DGAT1* p.K232A	201-250	KA	Heterozygote	582	29.76382	0.425544	28.92977	30.59787	a						
MilkYield	*DGAT1* p.K232A	201-250	AA	Mutant	859	31.2515	0.351496	30.56258	31.94042	b						
MilkYield	*DGAT1* p.K232A	251-300	KK	Wild	60	24.90343	1.242256	22.46866	27.33821	a						
MilkYield	*DGAT1* p.K232A	251-300	KA	Heterozygote	522	27.49451	0.43397	26.64394	28.34507	ab						
MilkYield	*DGAT1* p.K232A	251-300	AA	Mutant	762	28.2423	0.358959	27.53875	28.94585	b						
MilkYield	*DGAT1* p.K232A	301-350	KK	Wild	34	21.6664	1.426331	18.87085	24.46196	a						
MilkYield	*DGAT1* p.K232A	301-350	KA	Heterozygote	366	24.67627	0.467799	23.7594	25.59314	ab						
MilkYield	*DGAT1* p.K232A	301-350	AA	Mutant	444	25.45613	0.40491	24.66252	26.24974	b						
MilkYield	*DGAT1* p.K232A	351+	KK	Wild	27	21.03667	1.603456	17.89395	24.17938	-						
MilkYield	*DGAT1* p.K232A	351+	KA	Heterozygote	369	21.76129	0.474008	20.83225	22.69033	-						
MilkYield	*DGAT1* p.K232A	351+	AA	Mutant	477	21.95345	0.403458	21.16268	22.74421	-						
FatP	*GC-NPFFR2* rs137147462	0-50	AA	Wild	248	3.809956	0.058875	3.694563	3.925349	-	1.61E-294	0.97548317	0.02839632	1.83E-68	0.0516625	0.404522683
FatP	*GC-NPFFR2* rs137147462	0-50	AG	Heterozygote	687	3.832769	0.035496	3.763198	3.90234	-						

FatP	*GC-NPFFR2* rs137147462	0-50	GG	Mutant	447	3.888293	0.042698	3.804606	3.97198	-
FatP	*GC-NPFFR2* rs137147462	51-100	AA	Wild	289	3.589219	0.057697	3.476136	3.702303	-
FatP	*GC-NPFFR2* rs137147462	51-100	AG	Heterozygote	789	3.525433	0.034743	3.457338	3.593528	-
FatP	*GC-NPFFR2* rs137147462	51-100	GG	Mutant	546	3.515144	0.041385	3.434032	3.596257	-
FatP	*GC-NPFFR2* rs137147462	101-150	AA	Wild	284	3.660025	0.057736	3.546864	3.773186	-
FatP	*GC-NPFFR2* rs137147462	101-150	AG	Heterozygote	797	3.695689	0.034711	3.627657	3.763722	-
FatP	*GC-NPFFR2* rs137147462	101-150	GG	Mutant	513	3.659751	0.041766	3.57789	3.741611	-
FatP	*GC-NPFFR2* rs137147462	151-200	AA	Wild	278	3.83251	0.058	3.718831	3.946188	-
FatP	*GC-NPFFR2* rs137147462	151-200	AG	Heterozygote	752	3.839473	0.034975	3.770923	3.908024	-
FatP	*GC-NPFFR2* rs137147462	151-200	GG	Mutant	539	3.802561	0.041473	3.721274	3.883847	-
FatP	*GC-NPFFR2* rs137147462	201-250	AA	Wild	261	3.919431	0.058407	3.804955	4.033906	-
FatP	*GC-NPFFR2* rs137147462	201-250	AG	Heterozygote	734	3.935632	0.035095	3.866847	4.004417	-
FatP	*GC-NPFFR2* rs137147462	201-250	GG	Mutant	504	3.902874	0.041895	3.820762	3.984986	-
FatP	*GC-NPFFR2* rs137147462	251-300	AA	Wild	238	4.027084	0.059338	3.910785	4.143384	-
FatP	*GC-NPFFR2* rs137147462	251-300	AG	Heterozygote	660	4.054514	0.035609	3.984722	4.124307	-
FatP	*GC-NPFFR2* rs137147462	251-300	GG	Mutant	446	4.035002	0.042706	3.9513	4.118705	-
FatP	*GC-NPFFR2* rs137147462	301-350	AA	Wild	137	4.15286	0.065961	4.023578	4.282141	-
FatP	*GC-NPFFR2* rs137147462	301-350	AG	Heterozygote	414	4.159291	0.038853	4.083141	4.235441	-

	GC-NPFFR2				29	4.18689	0.04623									
FatP		301-350	GG	Mutant				4.096272	4.277512	-						
	rs137147462				3	2	5									
	GC-NPFFR2				11	4.30212										
FatP		351+	AA	Wild			0.06918	4.166537	4.437716	-						
	rs137147462				8	7										
	GC-NPFFR2				44	4.29238	0.03880									
FatP		351+	AG	Heterozygote				4.216335	4.368432	-						
	rs137147462				7	4	1									
	GC-NPFFR2				30	4.26098	0.04628									
FatP		351+	GG	Mutant				4.17027	4.351702	-						
	rs137147462				8	6	5									
	GC-NPFFR2				30	3.90441										
FatP		0-50	CC	Wild			0.05371	3.799147	4.009684	-						
	rs109452259				2	6										
	GC-NPFFR2				70	3.82857	0.03460									
FatP		0-50	CA	Heterozygote				3.760744	3.896398	-						
	rs109452259				6	1	6									
	GC-NPFFR2				37	3.83339	0.04706									
FatP		0-50	AA	Mutant				3.741154	3.925636	-						
	rs109452259				4	5	3									
	GC-NPFFR2				31	3.54828	0.05338									
FatP		51-100	CC	Wild				3.443652	3.652925	-						
	rs109452259				7	9	7									
	GC-NPFFR2				86	3.54984	0.03359									
FatP		51-100	CA	Heterozygote				3.483996	3.615686	-						
	rs109452259				2	1	5									
	GC-NPFFR2				44		0.04581									
FatP		51-100	AA	Mutant		3.49129		3.401496	3.581084	-						
	rs109452259				5		4									
	GC-NPFFR2				34	3.71219	0.05275									
FatP		101-150	CC	Wild				3.608794	3.81559	-						
	rs109452259				2	2	5				3.16E-	0.7436427	0.0335086		0.0480328	
														5.63E-69		0.029358315
	GC-NPFFR2				81						297	1	6		8	
FatP		101-150	CA	Heterozygote		3.6884	0.03387	3.622016	3.754783	-						
	rs109452259				1											
	GC-NPFFR2				44	3.62994	0.04590									
FatP		101-150	AA	Mutant				3.539963	3.719919	-						
	rs109452259				1	1	8									
	GC-NPFFR2				32		0.05318									
FatP		151-200	CC	Wild		3.83105		3.726816	3.935283	-						
	rs109452259				2		1									
	GC-NPFFR2				80	3.82198	0.03391									
FatP		151-200	CA	Heterozygote				3.755505	3.888459	-						
	rs109452259				5	2	8									
	GC-NPFFR2				44	3.82773	0.04587									
FatP		151-200	AA	Mutant				3.737819	3.917659	-						
	rs109452259				2	9	8									
	GC-NPFFR2				31	3.87856	0.05331									
FatP		201-250	CC	Wild				3.774067	3.983061	-						
	rs109452259				5	4	6									
	GC-NPFFR2				77	3.94273	0.03412									
FatP		201-250	CA	Heterozygote				3.875852	4.009615	-						
	rs109452259				2	3	4									

FatP	*GC-NPFFR2* rs109452259	201-250	AA	Mutant	412	3.915304	0.046348	3.824464	4.006144	-						
FatP	*GC-NPFFR2* rs109452259	251-300	CC	Wild	273	3.982917	0.054604	3.875894	4.089939	-						
FatP	*GC-NPFFR2* rs109452259	251-300	CA	Heterozygote	696	4.072438	0.034598	4.004628	4.140249	-						
FatP	*GC-NPFFR2* rs109452259	251-300	AA	Mutant	375	4.032052	0.047044	3.939849	4.124256	-						
FatP	*GC-NPFFR2* rs109452259	301-350	CC	Wild	150	4.088902	0.061501	3.968362	4.209441	-						
FatP	*GC-NPFFR2* rs109452259	301-350	CA	Heterozygote	422	4.194595	0.038059	4.120001	4.269189	-						
FatP	*GC-NPFFR2* rs109452259	301-350	AA	Mutant	272	4.166845	0.049781	4.069275	4.264415	-						
FatP	*GC-NPFFR2* rs109452259	351+	CC	Wild	153	4.224282	0.062063	4.10264	4.345923	-						
FatP	*GC-NPFFR2* rs109452259	351+	CA	Heterozygote	445	4.319162	0.038242	4.24421	4.394115	-						
FatP	*GC-NPFFR2* rs109452259	351+	AA	Mutant	275	4.253891	0.050193	4.155515	4.352266	-						
FatP	*BRCA1* rs134817801	0-50	AA	Wild	443	3.857446	0.04265	3.773854	3.941038	-						
FatP	*BRCA1* rs134817801	0-50	AC	Heterozygote	670	3.81279	0.035271	3.74366	3.881919	-						
FatP	*BRCA1* rs134817801	0-50	CC	Mutant	269	3.914009	0.056934	3.80242	4.025597	-						
FatP	*BRCA1* rs134817801	51-100	AA	Wild	527	3.548944	0.041504	3.467597	3.630292	-						
											0	0.01632314	0.0242591	9.87E-69	0.06986299	0.076603624
FatP	*BRCA1* rs134817801	51-100	AC	Heterozygote	788	3.497469	0.034405	3.430036	3.564902	-						
FatP	*BRCA1* rs134817801	51-100	CC	Mutant	309	3.597456	0.05578	3.48813	3.706783	-						
FatP	*BRCA1* rs134817801	101-150	AA	Wild	512	3.698039	0.041678	3.616351	3.779727	-						
FatP	*BRCA1* rs134817801	101-150	AC	Heterozygote	768	3.631538	0.034524	3.563872	3.699203	-						

FatP	*BRCA1* rs134817801	101-150	CC	Mutant	314	3.759746	0.055682	3.65061	3.868881	-							
FatP	*BRCA1* rs134817801	151-200	AA	Wild	491	3.86255	0.041894	3.78044	3.94466	-							
FatP	*BRCA1* rs134817801	151-200	AC	Heterozygote	760	3.770044	0.034595	3.70224	3.837848	-							
FatP	*BRCA1* rs134817801	151-200	CC	Mutant	318	3.906165	0.055573	3.797244	4.015086	-							
FatP	*BRCA1* rs134817801	201-250	AA	Wild	490	3.957327	0.041876	3.875251	4.039403	ab							
FatP	*BRCA1* rs134817801	201-250	AC	Heterozygote	702	3.849864	0.035006	3.781255	3.918474	a							
FatP	*BRCA1* rs134817801	201-250	CC	Mutant	307	4.039474	0.055842	3.930026	4.148922	b							
FatP	*BRCA1* rs134817801	251-300	AA	Wild	432	4.030951	0.042687	3.947286	4.114617	-							
FatP	*BRCA1* rs134817801	251-300	AC	Heterozygote	649	4.012874	0.035424	3.943443	4.082304	-							
FatP	*BRCA1* rs134817801	251-300	CC	Mutant	263	4.140322	0.05715	4.028309	4.252334	-							
FatP	*BRCA1* rs134817801	301-350	AA	Wild	271	4.169153	0.046789	4.077448	4.260858	ab							
FatP	*BRCA1* rs134817801	301-350	AC	Heterozygote	427	4.1123	0.038346	4.037143	4.187457	a							
FatP	*BRCA1* rs134817801	301-350	CC	Mutant	146	4.314447	0.063915	4.189176	4.439717	b							
FatP	*BRCA1* rs134817801	351+	AA	Wild	314	4.294252	0.046235	4.203633	4.384872	a							
FatP	*BRCA1* rs134817801	351+	AC	Heterozygote	422	4.193222	0.038804	4.117167	4.269277	a							
FatP	*BRCA1* rs134817801	351+	CC	Mutant	137	4.514196	0.065715	4.385397	4.642996	b							
FatP	*DGAT1* p.K232A	0-50	KK	Wild	68	4.324106	0.093776	4.140308	4.507905	c							
FatP	*DGAT1* p.K232A	0-50	KA	Heterozygote	538	3.944451	0.033303	3.879179	4.009724	b							
FatP	*DGAT1* p.K232A	0-50	AA	Mutant	776	3.739734	0.02759	3.68566	3.793809	a							
FatP	*DGAT1* p.K232A	51-100	KK	Wild	68	4.032737	0.093596	3.849292	4.216182	c							
FatP	*DGAT1* p.K232A	51-100	KA	Heterozygote	619	3.705289	0.032247	3.642087	3.768492	b							
FatP	*DGAT1* p.K232A	51-100	AA	Mutant	937	3.371798	0.026521	3.319817	3.423779	a							
FatP	*DGAT1* p.K232A	101-150	KK	Wild	65	4.301002	0.094567	4.115655	4.48635	c							
FatP	*DGAT1* p.K232A	101-150	KA	Heterozygote	616	3.846853	0.032333	3.783482	3.910225	b							
FatP	*DGAT1* p.K232A	101-150	AA	Mutant	913	3.509146	0.026635	3.456942	3.56135	a							
FatP	*DGAT1* p.K232A	151-200	KK	Wild	71	4.377702	0.092591	4.196226	4.559177	c	1.16E-141	3.48E-32	0.03361081	2.45E-71	0.01157071		1.57E-08
FatP	*DGAT1* p.K232A	151-200	KA	Heterozygote	628	4.03093	0.032175	3.967869	4.093991	b							
FatP	*DGAT1* p.K232A	151-200	AA	Mutant	870	3.636123	0.026915	3.583371	3.688875	a							
FatP	*DGAT1* p.K232A	201-250	KK	Wild	58	4.428962	0.097148	4.238556	4.619369	c							
FatP	*DGAT1* p.K232A	201-250	KA	Heterozygote	582	4.124427	0.032676	4.060382	4.188471	b							
FatP	*DGAT1* p.K232A	201-250	AA	Mutant	859	3.738765	0.026976	3.685894	3.791637	a							
FatP	*DGAT1* p.K232A	251-300	KK	Wild	60	4.74412	0.096036	4.555894	4.932347	c							
FatP	*DGAT1* p.K232A	251-300	KA	Heterozygote	522	4.242021	0.033457	4.176447	4.307595	b							
FatP	*DGAT1* p.K232A	251-300	AA	Mutant	762	3.847113	0.027669	3.792882	3.901344	a							
FatP	*DGAT1* p.K232A	301-350	KK	Wild	34	4.950296	0.112742	4.729326	5.171267	c							

FatP	*DGAT1* p.K232A	301-350	KA	Heterozygote	366	4.393646	0.036557	4.321996	4.465295	b						
FatP	*DGAT1* p.K232A	301-350	AA	Mutant	444	3.938854	0.031862	3.876406	4.001303	a						
FatP	*DGAT1* p.K232A	351+	KK	Wild	27	5.116673	0.128182	4.865442	5.367905	c						
FatP	*DGAT1* p.K232A	351+	KA	Heterozygote	369	4.49333	0.037095	4.420626	4.566034	b						
FatP	*DGAT1* p.K232A	351+	AA	Mutant	477	4.069164	0.031716	4.007001	4.131326	a						
ProteinP	*GC-NPFFR2* rs137147462	0-50	AA	Wild	248	3.203272	0.032453	3.139666	3.266878	-						
ProteinP	*GC-NPFFR2* rs137147462	0-50	AG	Heterozygote	687	3.182839	0.019614	3.144395	3.221283	-						
ProteinP	*GC-NPFFR2* rs137147462	0-50	GG	Mutant	447	3.138733	0.023504	3.092665	3.1848	-						
ProteinP	*GC-NPFFR2* rs137147462	51-100	AA	Wild	289	3.206741	0.032152	3.143725	3.269757	b						
ProteinP	*GC-NPFFR2* rs137147462	51-100	AG	Heterozygote	789	3.156222	0.019408	3.118184	3.19426	ab						
ProteinP	*GC-NPFFR2* rs137147462	51-100	GG	Mutant	546	3.106768	0.023145	3.061405	3.15213	a						
ProteinP	*GC-NPFFR2* rs137147462	101-150	AA	Wild	284	3.358334	0.032158	3.295305	3.421363	-						
ProteinP	*GC-NPFFR2* rs137147462	101-150	AG	Heterozygote	797	3.321455	0.0194	3.283432	3.359477	-						
ProteinP	*GC-NPFFR2* rs137147462	101-150	GG	Mutant	513	3.271148	0.023248	3.225583	3.316714	-						
ProteinP	*GC-NPFFR2* rs137147462	151-200	AA	Wild	278	3.444253	0.032232	3.38108	3.507426	-						
ProteinP	*GC-NPFFR2* rs137147462	151-200	AG	Heterozygote	752	3.395605	0.01947	3.357444	3.433766	-						
ProteinP	*GC-NPFFR2* rs137147462	151-200	GG	Mutant	539	3.368482	0.023169	3.323072	3.413892	-						
											0	0.20212075	2.79E-20	2.75E-188	1.12E-30	0.081037855
ProteinP	*GC-NPFFR2* rs137147462	201-250	AA	Wild	261	3.500102	0.03234	3.436717	3.563486	-						
ProteinP	*GC-NPFFR2* rs137147462	201-250	AG	Heterozygote	734	3.463615	0.019503	3.42539	3.50184	-						
ProteinP	*GC-NPFFR2* rs137147462	201-250	GG	Mutant	504	3.450249	0.023284	3.404613	3.495885	-						
ProteinP	*GC-NPFFR2* rs137147462	251-300	AA	Wild	238	3.590702	0.032598	3.526812	3.654592	-						
ProteinP	*GC-NPFFR2* rs137147462	251-300	AG	Heterozygote	660	3.556916	0.01964	3.518423	3.59541	-						
ProteinP	*GC-NPFFR2* rs137147462	251-300	GG	Mutant	446	3.536701	0.023506	3.49063	3.582772	-						
ProteinP	*GC-NPFFR2* rs137147462	301-350	AA	Wild	137	3.712738	0.034471	3.645177	3.780299	-						
ProteinP	*GC-NPFFR2* rs137147462	301-350	AG	Heterozygote	414	3.657392	0.02055	3.617114	3.69767	-						
ProteinP	*GC-NPFFR2* rs137147462	301-350	GG	Mutant	293	3.631738	0.024496	3.583727	3.679748	-						
ProteinP	*GC-NPFFR2* rs137147462	351+	AA	Wild	118	3.778686	0.035424	3.709257	3.848115	-						
ProteinP	*GC-NPFFR2* rs137147462	351+	AG	Heterozygote	447	3.788537	0.020543	3.748274	3.8288	-						
ProteinP	*GC-NPFFR2* rs137147462	351+	GG	Mutant	308	3.767084	0.024516	3.719033	3.815136	-						
ProteinP	*GC-NPFFR2* rs109452259	0-50	CC	Wild	302	3.207253	0.029735	3.148973	3.265534	-						
ProteinP	*GC-NPFFR2* rs109452259	0-50	CA	Heterozygote	706	3.169612	0.019116	3.132145	3.207078	-						
ProteinP	*GC-NPFFR2* rs109452259	0-50	AA	Mutant	374	3.14628	0.026032	3.095257	3.197302	-						
ProteinP	*GC-NPFFR2* rs109452259	51-100	CC	Wild	317	3.160692	0.029662	3.102556	3.218828	-	0	0.40497979	6.49E-20	5.42E-189	3.19E-30	1.05E-06
ProteinP	*GC-NPFFR2* rs109452259	51-100	CA	Heterozygote	862	3.164087	0.018841	3.127159	3.201015	-						
ProteinP	*GC-NPFFR2* rs109452259	51-100	AA	Mutant	445	3.109452	0.025692	3.059097	3.159808	-						
ProteinP	*GC-NPFFR2* rs109452259	101-150	CC	Wild	342	3.322855	0.029489	3.265057	3.380654	-						

ProteinP	*GC-NPFFR2* rs109452259	101-150	CA	Heterozygote	811	3.321772	0.018914	3.284701	3.358842	-						
ProteinP	*GC-NPFFR2* rs109452259	101-150	AA	Mutant	441	3.281743	0.025719	3.231335	3.332151	-						
ProteinP	*GC-NPFFR2* rs109452259	151-200	CC	Wild	322	3.409624	0.029603	3.351604	3.467644	-						
ProteinP	*GC-NPFFR2* rs109452259	151-200	CA	Heterozygote	805	3.406329	0.018927	3.369232	3.443426	-						
ProteinP	*GC-NPFFR2* rs109452259	151-200	AA	Mutant	442	3.362676	0.02571	3.312285	3.413068	-						
ProteinP	*GC-NPFFR2* rs109452259	201-250	CC	Wild	315	3.451275	0.029638	3.393185	3.509365	-						
ProteinP	*GC-NPFFR2* rs109452259	201-250	CA	Heterozygote	772	3.479587	0.018984	3.442378	3.516796	-						
ProteinP	*GC-NPFFR2* rs109452259	201-250	AA	Mutant	412	3.449372	0.025836	3.398734	3.500011	-						
ProteinP	*GC-NPFFR2* rs109452259	251-300	CC	Wild	273	3.538236	0.029993	3.479451	3.59702	-						
ProteinP	*GC-NPFFR2* rs109452259	251-300	CA	Heterozygote	696	3.569909	0.019109	3.532457	3.607361	-						
ProteinP	*GC-NPFFR2* rs109452259	251-300	AA	Mutant	375	3.542137	0.026027	3.491125	3.593149	-						
ProteinP	*GC-NPFFR2* rs109452259	301-350	CC	Wild	150	3.61988	0.031949	3.557261	3.682498	-						
ProteinP	*GC-NPFFR2* rs109452259	301-350	CA	Heterozygote	422	3.681623	0.020079	3.642269	3.720976	-						
ProteinP	*GC-NPFFR2* rs109452259	301-350	AA	Mutant	272	3.637734	0.026783	3.585241	3.690228	-						
ProteinP	*GC-NPFFR2* rs109452259	351+	CC	Wild	153	3.709407	0.032123	3.646447	3.772367	a						
ProteinP	*GC-NPFFR2* rs109452259	351+	CA	Heterozygote	445	3.814533	0.020141	3.775058	3.854007	b						
ProteinP	*GC-NPFFR2* rs109452259	351+	AA	Mutant	275	3.76422	0.026906	3.711486	3.816955	ab						
ProteinP	*BRCA1* rs134817801	0-50	AA	Wild	443	3.173441	0.023474	3.127433	3.21945	-						
ProteinP	*BRCA1* rs134817801	0-50	AC	Heterozygote	670	3.166482	0.019605	3.128057	3.204906	-						
ProteinP	*BRCA1* rs134817801	0-50	CC	Mutant	269	3.178121	0.031871	3.115656	3.240586	-						
ProteinP	*BRCA1* rs134817801	51-100	AA	Wild	527	3.170233	0.023162	3.124836	3.215629	-						
ProteinP	*BRCA1* rs134817801	51-100	AC	Heterozygote	788	3.121227	0.019374	3.083255	3.159199	-						
ProteinP	*BRCA1* rs134817801	51-100	CC	Mutant	309	3.176206	0.03156	3.11435	3.238062	-						
ProteinP	*BRCA1* rs134817801	101-150	AA	Wild	512	3.338605	0.023208	3.293119	3.384092	-						
ProteinP	*BRCA1* rs134817801	101-150	AC	Heterozygote	768	3.287396	0.019406	3.249362	3.325431	-						
ProteinP	*BRCA1* rs134817801	101-150	CC	Mutant	314	3.321928	0.031535	3.26012	3.383736	-						
ProteinP	*BRCA1* rs134817801	151-200	AA	Wild	491	3.42336	0.023264	3.377762	3.468957	-	0	0.04663265	1.21E-19	1.56E-185	1.19E-29	2.18E-06
ProteinP	*BRCA1* rs134817801	151-200	AC	Heterozygote	760	3.366689	0.019425	3.328617	3.404761	-						
ProteinP	*BRCA1* rs134817801	151-200	CC	Mutant	318	3.416	0.031506	3.35425	3.47775	-						
ProteinP	*BRCA1* rs134817801	201-250	AA	Wild	490	3.486528	0.023258	3.440943	3.532112	-						
ProteinP	*BRCA1* rs134817801	201-250	AC	Heterozygote	702	3.432461	0.019537	3.394169	3.470752	-						
ProteinP	*BRCA1* rs134817801	201-250	CC	Mutant	307	3.508471	0.031577	3.446581	3.57036	-						
ProteinP	*BRCA1* rs134817801	251-300	AA	Wild	432	3.586717	0.023477	3.540703	3.63273	-						
ProteinP	*BRCA1* rs134817801	251-300	AC	Heterozygote	649	3.515282	0.019648	3.476772	3.553792	-						
ProteinP	*BRCA1* rs134817801	251-300	CC	Mutant	263	3.602881	0.03193	3.5403	3.665462	-						
ProteinP	*BRCA1* rs134817801	301-350	AA	Wild	271	3.700134	0.024627	3.651866	3.748402	b						

ProteinP	*BRCA1* rs134817801	301-350	AC	Heterozygote	427	3.609495	0.020461	3.569392	3.649598	a						
ProteinP	*BRCA1* rs134817801	301-350	CC	Mutant	146	3.712017	0.033814	3.645744	3.778291	b						
ProteinP	*BRCA1* rs134817801	351+	AA	Wild	314	3.818428	0.024482	3.770445	3.866411	b						
ProteinP	*BRCA1* rs134817801	351+	AC	Heterozygote	422	3.726682	0.020596	3.686314	3.76705	a						
ProteinP	*BRCA1* rs134817801	351+	CC	Mutant	137	3.862818	0.034342	3.79551	3.930127	b						
ProteinP	*DGAT1* p.K232A	0-50	KK	Wild	68	3.320489	0.056694	3.209372	3.431607	b						
ProteinP	*DGAT1* p.K232A	0-50	KA	Heterozygote	538	3.218074	0.020096	3.178686	3.257462	b						
ProteinP	*DGAT1* p.K232A	0-50	AA	Mutant	776	3.126355	0.016637	3.093747	3.158963	a						
ProteinP	*DGAT1* p.K232A	51-100	KK	Wild	68	3.316375	0.056643	3.205357	3.427393	b						
ProteinP	*DGAT1* p.K232A	51-100	KA	Heterozygote	619	3.222333	0.019828	3.18347	3.261195	b						
ProteinP	*DGAT1* p.K232A	51-100	AA	Mutant	937	3.081328	0.016378	3.049228	3.113428	a						
ProteinP	*DGAT1* p.K232A	101-150	KK	Wild	65	3.461077	0.056887	3.349581	3.572573	b						
ProteinP	*DGAT1* p.K232A	101-150	KA	Heterozygote	616	3.406813	0.019853	3.367902	3.445724	b						
ProteinP	*DGAT1* p.K232A	101-150	AA	Mutant	913	3.231017	0.016403	3.198868	3.263166	a						
ProteinP	*DGAT1* p.K232A	151-200	KK	Wild	71	3.57122	0.056387	3.460703	3.681736	b						
ProteinP	*DGAT1* p.K232A	151-200	KA	Heterozygote	628	3.492979	0.019814	3.454145	3.531813	b						
ProteinP	*DGAT1* p.K232A	151-200	AA	Mutant	870	3.310864	0.016471	3.278581	3.343148	a	0	1.43E-13	3.47E-20	1.23E-193	6.64E-30	2.70E-14
ProteinP	*DGAT1* p.K232A	201-250	KK	Wild	58	3.662466	0.057545	3.54968	3.775251	b						
ProteinP	*DGAT1* p.K232A	201-250	KA	Heterozygote	582	3.565318	0.019935	3.526246	3.604391	b						
ProteinP	*DGAT1* p.K232A	201-250	AA	Mutant	859	3.378985	0.016487	3.346671	3.4113	a						
ProteinP	*DGAT1* p.K232A	251-300	KK	Wild	60	3.791269	0.057248	3.679065	3.903474	b						
ProteinP	*DGAT1* p.K232A	251-300	KA	Heterozygote	522	3.662118	0.020128	3.622667	3.701568	b						
ProteinP	*DGAT1* p.K232A	251-300	AA	Mutant	762	3.462222	0.016656	3.429576	3.494868	a						
ProteinP	*DGAT1* p.K232A	301-350	KK	Wild	34	3.862872	0.061696	3.74195	3.983794	b						
ProteinP	*DGAT1* p.K232A	301-350	KA	Heterozygote	366	3.762365	0.020925	3.721353	3.803377	b						
ProteinP	*DGAT1* p.K232A	301-350	AA	Mutant	444	3.565514	0.017752	3.530721	3.600307	a						
ProteinP	*DGAT1* p.K232A	351+	KK	Wild	27	4.137932	0.066418	4.007755	4.268108	c						
ProteinP	*DGAT1* p.K232A	351+	KA	Heterozygote	369	3.87059	0.021089	3.829257	3.911924	b						
ProteinP	*DGAT1* p.K232A	351+	AA	Mutant	477	3.694057	0.017723	3.659321	3.728793	a						
SNFP	*GC-NPFFR2* rs137147462	0-50	AA	Wild	248	8.731539	0.039581	8.653963	8.809116	-						
SNFP	*GC-NPFFR2* rs137147462	0-50	AG	Heterozygote	687	8.707732	0.02393	8.66083	8.754634	-						
SNFP	*GC-NPFFR2* rs137147462	0-50	GG	Mutant	447	8.625706	0.028668	8.569518	8.681893	-						
SNFP	*GC-NPFFR2* rs137147462	51-100	AA	Wild	289	8.774254	0.039248	8.697329	8.851179	b	0	0.04870887	6.39E-07	1.16E-117	5.24E-41	0.142902066
SNFP	*GC-NPFFR2* rs137147462	51-100	AG	Heterozygote	789	8.750103	0.023699	8.703653	8.796553	b						
SNFP	*GC-NPFFR2* rs137147462	51-100	GG	Mutant	546	8.648482	0.028267	8.593079	8.703885	a						
SNFP	*GC-NPFFR2* rs137147462	101-150	AA	Wild	284	8.897126	0.039255	8.820188	8.974064	ab						

SNFP	*GC-NPFFR2* rs137147462	101-150	AG	Heterozygote	797	8.879186	0.023691	8.832753	8.925618	b						
SNFP	*GC-NPFFR2* rs137147462	101-150	GG	Mutant	513	8.788192	0.028382	8.732563	8.84382	a						
SNFP	*GC-NPFFR2* rs137147462	151-200	AA	Wild	278	8.932692	0.039337	8.855593	9.009792	-						
SNFP	*GC-NPFFR2* rs137147462	151-200	AG	Heterozygote	752	8.913201	0.023769	8.866614	8.959788	-						
SNFP	*GC-NPFFR2* rs137147462	151-200	GG	Mutant	539	8.850692	0.028294	8.795237	8.906147	-						
SNFP	*GC-NPFFR2* rs137147462	201-250	AA	Wild	261	8.954743	0.039457	8.877409	9.032077	-						
SNFP	*GC-NPFFR2* rs137147462	201-250	AG	Heterozygote	734	8.934318	0.023805	8.88766	8.980975	-						
SNFP	*GC-NPFFR2* rs137147462	201-250	GG	Mutant	504	8.888686	0.028422	8.832979	8.944393	-						
SNFP	*GC-NPFFR2* rs137147462	251-300	AA	Wild	238	9.020196	0.039745	8.942298	9.098094	-						
SNFP	*GC-NPFFR2* rs137147462	251-300	AG	Heterozygote	660	9.002817	0.023958	8.955861	9.049774	-						
SNFP	*GC-NPFFR2* rs137147462	251-300	GG	Mutant	446	8.941602	0.02867	8.885411	8.997794	-						
SNFP	*GC-NPFFR2* rs137147462	301-350	AA	Wild	137	9.146857	0.041837	9.064858	9.228856	b						
SNFP	*GC-NPFFR2* rs137147462	301-350	AG	Heterozygote	414	9.082616	0.024974	9.033668	9.131564	ab						
SNFP	*GC-NPFFR2* rs137147462	301-350	GG	Mutant	293	9.013823	0.029774	8.955467	9.07218	a						
SNFP	*GC-NPFFR2* rs137147462	351+	AA	Wild	118	9.189647	0.042907	9.105551	9.273742	-						
SNFP	*GC-NPFFR2* rs137147462	351+	AG	Heterozygote	447	9.197612	0.024966	9.148678	9.246545	-						
SNFP	*GC-NPFFR2* rs137147462	351+	GG	Mutant	308	9.121602	0.029798	9.063199	9.180005	-						
SNFP	*GC-NPFFR2* rs109452259	0-50	CC	Wild	302	8.727716	0.036284	8.656602	8.798831	-						
SNFP	*GC-NPFFR2* rs109452259	0-50	CA	Heterozygote	706	8.697606	0.023323	8.651895	8.743318	-						
SNFP	*GC-NPFFR2* rs109452259	0-50	AA	Mutant	374	8.623951	0.031767	8.56169	8.686213	-						
SNFP	*GC-NPFFR2* rs109452259	51-100	CC	Wild	317	8.731592	0.036204	8.660634	8.802551	ab						
SNFP	*GC-NPFFR2* rs109452259	51-100	CA	Heterozygote	862	8.751154	0.023017	8.706042	8.796265	b						
SNFP	*GC-NPFFR2* rs109452259	51-100	AA	Mutant	445	8.652693	0.031388	8.591174	8.714213	a						
SNFP	*GC-NPFFR2* rs109452259	101-150	CC	Wild	342	8.862613	0.036012	8.792031	8.933195	-						
SNFP	*GC-NPFFR2* rs109452259	101-150	CA	Heterozygote	811	8.879594	0.023097	8.834324	8.924864	-						
SNFP	*GC-NPFFR2* rs109452259	101-150	AA	Mutant	441	8.790893	0.031418	8.729316	8.85247	-						
SNFP	*GC-NPFFR2* rs109452259	151-200	CC	Wild	322	8.904434	0.036138	8.833605	8.975262	-	0	0.08461747	4.32E-07	3.04E-118	1.12E-40	7.71E-06
SNFP	*GC-NPFFR2* rs109452259	151-200	CA	Heterozygote	805	8.917542	0.023112	8.872242	8.962841	-						
SNFP	*GC-NPFFR2* rs109452259	151-200	AA	Mutant	442	8.847178	0.031408	8.785619	8.908737	-						
SNFP	*GC-NPFFR2* rs109452259	201-250	CC	Wild	315	8.896286	0.036177	8.82538	8.967192	-						
SNFP	*GC-NPFFR2* rs109452259	201-250	CA	Heterozygote	772	8.95098	0.023176	8.905556	8.996404	-						
SNFP	*GC-NPFFR2* rs109452259	201-250	AA	Mutant	412	8.888416	0.031549	8.826583	8.95025	-						
SNFP	*GC-NPFFR2* rs109452259	251-300	CC	Wild	273	8.948708	0.036572	8.877028	9.020388	-						
SNFP	*GC-NPFFR2* rs109452259	251-300	CA	Heterozygote	696	9.021003	0.023314	8.975309	9.066698	-						
SNFP	*GC-NPFFR2* rs109452259	251-300	AA	Mutant	375	8.943959	0.031761	8.881709	9.006209	-						
SNFP	*GC-NPFFR2* rs109452259	301-350	CC	Wild	150	9.044018	0.038759	8.968052	9.119985	-						

SNFP	*GC-NPFFR2* rs109452259	301-350	CA	Heterozygote	422	9.108212	0.024397	9.060394	9.15603	-						
SNFP	*GC-NPFFR2* rs109452259	301-350	AA	Mutant	272	9.015961	0.032604	8.952058	9.079863	-						
SNFP	*GC-NPFFR2* rs109452259	351+	CC	Wild	153	9.103814	0.038955	9.027463	9.180165	a						
SNFP	*GC-NPFFR2* rs109452259	351+	CA	Heterozygote	445	9.216894	0.024467	9.168939	9.264848	b						
SNFP	*GC-NPFFR2* rs109452259	351+	AA	Mutant	275	9.128837	0.032742	9.064665	9.193009	ab						
SNFP	*BRCA1* rs134817801	0-50	AA	Wild	443	8.675989	0.028726	8.619687	8.732292	-						
SNFP	*BRCA1* rs134817801	0-50	AC	Heterozygote	670	8.682543	0.024015	8.635474	8.729611	-						
SNFP	*BRCA1* rs134817801	0-50	CC	Mutant	269	8.698176	0.039068	8.621604	8.774748	-						
SNFP	*BRCA1* rs134817801	51-100	AA	Wild	527	8.742602	0.02838	8.686978	8.798225	-						
SNFP	*BRCA1* rs134817801	51-100	AC	Heterozygote	788	8.68954	0.02376	8.642971	8.736108	-						
SNFP	*BRCA1* rs134817801	51-100	CC	Mutant	309	8.753277	0.038724	8.677379	8.829174	-						
SNFP	*BRCA1* rs134817801	101-150	AA	Wild	512	8.880285	0.028431	8.824562	8.936009	-						
SNFP	*BRCA1* rs134817801	101-150	AC	Heterozygote	768	8.821097	0.023795	8.77446	8.867735	-						
SNFP	*BRCA1* rs134817801	101-150	CC	Mutant	314	8.876842	0.038697	8.800997	8.952687	-						
SNFP	*BRCA1* rs134817801	151-200	AA	Wild	491	8.927212	0.028493	8.871366	8.983059	-						
SNFP	*BRCA1* rs134817801	151-200	AC	Heterozygote	760	8.861385	0.023816	8.814707	8.908064	-						
SNFP	*BRCA1* rs134817801	151-200	CC	Mutant	318	8.924168	0.038664	8.848388	8.999948	-	0	0.0543227	1.80E-07	1.56E-115	8.29E-40	8.28E-08
SNFP	*BRCA1* rs134817801	201-250	AA	Wild	490	8.949413	0.028486	8.893582	9.005245	-						
SNFP	*BRCA1* rs134817801	201-250	AC	Heterozygote	702	8.87928	0.02394	8.832358	8.926202	-						
SNFP	*BRCA1* rs134817801	201-250	CC	Mutant	307	8.978821	0.038743	8.902887	9.054756	-						
SNFP	*BRCA1* rs134817801	251-300	AA	Wild	432	9.026027	0.028728	8.96972	9.082333	b						
SNFP	*BRCA1* rs134817801	251-300	AC	Heterozygote	649	8.933888	0.024064	8.886724	8.981052	a						
SNFP	*BRCA1* rs134817801	251-300	CC	Mutant	263	9.04019	0.039134	8.963489	9.11689	ab						
SNFP	*BRCA1* rs134817801	301-350	AA	Wild	271	9.122655	0.030007	9.063842	9.181468	b						
SNFP	*BRCA1* rs134817801	301-350	AC	Heterozygote	427	9.008399	0.024967	8.959465	9.057332	a						
SNFP	*BRCA1* rs134817801	301-350	CC	Mutant	146	9.139449	0.041227	9.058647	9.220252	b						
SNFP	*BRCA1* rs134817801	351+	AA	Wild	314	9.233051	0.029846	9.174553	9.291548	b						
SNFP	*BRCA1* rs134817801	351+	AC	Heterozygote	422	9.107124	0.025117	9.057895	9.156353	a						
SNFP	*BRCA1* rs134817801	351+	CC	Mutant	137	9.227829	0.041816	9.145872	9.309786	b						
SNFP	*DGAT1* p.K232A	0-50	KK	Wild	68	8.843719	0.070093	8.706339	8.981099	b						
SNFP	*DGAT1* p.K232A	0-50	KA	Heterozygote	538	8.749286	0.02484	8.7006	8.797972	b						
SNFP	*DGAT1* p.K232A	0-50	AA	Mutant	776	8.625263	0.020563	8.58496	8.665566	a						
SNFP	*DGAT1* p.K232A	51-100	KK	Wild	68	8.894418	0.070037	8.757148	9.031687	b	9.49E-294	4.65E-12	5.80E-08	2.66E-121	7.04E-40	1.36E-09
SNFP	*DGAT1* p.K232A	51-100	KA	Heterozygote	619	8.817287	0.024545	8.769181	8.865394	b						
SNFP	*DGAT1* p.K232A	51-100	AA	Mutant	937	8.636034	0.020279	8.596289	8.67578	a						
SNFP	*DGAT1* p.K232A	101-150	KK	Wild	65	9.039362	0.070306	8.901566	9.177159	b						

SNFP	*DGAT1* p.K232A	101-150	KA	Heterozygote	616	8.969374	0.024572	8.921214	9.017534	b
SNFP	*DGAT1* p.K232A	101-150	AA	Mutant	913	8.753374	0.020306	8.713574	8.793173	a
SNFP	*DGAT1* p.K232A	151-200	KK	Wild	71	9.10187	0.069754	8.965154	9.238586	b
SNFP	*DGAT1* p.K232A	151-200	KA	Heterozygote	628	9.011665	0.024529	8.963589	9.059741	b
SNFP	*DGAT1* p.K232A	151-200	AA	Mutant	870	8.79602	0.020382	8.756073	8.835967	a
SNFP	*DGAT1* p.K232A	201-250	KK	Wild	58	9.159024	0.071031	9.019806	9.298243	b
SNFP	*DGAT1* p.K232A	201-250	KA	Heterozygote	582	9.033289	0.024663	8.984951	9.081627	b
SNFP	*DGAT1* p.K232A	201-250	AA	Mutant	859	8.824949	0.020399	8.784968	8.864931	a
SNFP	*DGAT1* p.K232A	251-300	KK	Wild	60	9.267709	0.070703	9.129133	9.406285	b
SNFP	*DGAT1* p.K232A	251-300	KA	Heterozygote	522	9.097012	0.024875	9.048258	9.145766	b
SNFP	*DGAT1* p.K232A	251-300	AA	Mutant	762	8.883379	0.020585	8.843033	8.923725	a
SNFP	*DGAT1* p.K232A	301-350	KK	Wild	34	9.308589	0.075629	9.160359	9.456818	b
SNFP	*DGAT1* p.K232A	301-350	KA	Heterozygote	366	9.185572	0.025755	9.135094	9.23605	b
SNFP	*DGAT1* p.K232A	301-350	AA	Mutant	444	8.967304	0.021796	8.924584	9.010024	a
SNFP	*DGAT1* p.K232A	351+	KK	Wild	27	9.584892	0.080914	9.426303	9.743481	c
SNFP	*DGAT1* p.K232A	351+	KA	Heterozygote	369	9.271985	0.025938	9.221149	9.322822	b
SNFP	*DGAT1* p.K232A	351+	AA	Mutant	477	9.072872	0.021765	9.030214	9.11553	a

Genotype effects were estimated for four SNPs across five milk-related variables:SCS, Test-day Milk Yield in kilograms (Milk Yield), Test-day Fat Percentage (FatP), Test-day Protein Percentage (ProteinP), and Test-day Solids-not-fat Percentage (SNFP).Linear mixed models included DIM stage, parity group, and season as fixed effects, and cow ID as a random effect. LSMs ± SEs, 95% confidence intervals (Lower CI, Upper CI), and Tukey-adjusted groupings (Tukey group) are presented for each genotype within DIM stage. P-values are shown for Genotype, DIM stage, Parity group, Season, and the interaction between Genotype and DIM stage. *p*-values reported as "$p < 1.0e\text{-}300$" indicate values below the computational detection threshold.

Table S4. Full results of inheritance-model comparisons for all SNP–trait combinations

Trait	SNP	Inheritance model	*p* value	AIC	genotype	LSM	SE
SCS	*GC-NPFFR2* rs137147462	additive	0.2082	42432.4	AA	2.315	0.136
SCS	*GC-NPFFR2* rs137147462	additive	0.2082	42432.4	AG	2.514	0.082
SCS	*GC-NPFFR2* rs137147462	additive	0.2082	42432.4	GG	2.798	0.097
SCS	*GC-NPFFR2* rs137147462	dominant	0.03713	42435.4	AA	2.316	0.138
SCS	*GC-NPFFR2* rs137147462	dominant	0.03713	42435.4	AG+GG	2.631	0.063
SCS	*GC-NPFFR2* rs137147462	recessive	0.005409	42432	AA+AG	2.462	0.071
SCS	*GC-NPFFR2* rs137147462	recessive	0.005409	42432	GG	2.798	0.097
SCS	*GC-NPFFR2* rs137147462	overdominant	0.2994	42438.7	AA+GG	2.635	0.08
SCS	*GC-NPFFR2* rs137147462	overdominant	0.2994	42438.7	AG	2.516	0.083

SCS	*GC-NPFFR2* rs109452259	additive	0.7583	42441.1	CC	2.582	0.127
SCS	*GC-NPFFR2* rs109452259	additive	0.7583	42441.1	CA	2.536	0.081
SCS	*GC-NPFFR2* rs109452259	additive	0.7583	42441.1	AA	2.648	0.109
SCS	*GC-NPFFR2* rs109452259	dominant	0.9597	42439.8	CC	2.583	0.127
SCS	*GC-NPFFR2* rs109452259	dominant	0.9597	42439.8	CA+AA	2.576	0.065
SCS	*GC-NPFFR2* rs109452259	recessive	0.4456	42439.2	CC+CA	2.55	0.069
SCS	*GC-NPFFR2* rs109452259	recessive	0.4456	42439.2	AA	2.648	0.109
SCS	*GC-NPFFR2* rs109452259	overdominant	0.4696	42439.3	CC+AA	2.62	0.083
SCS	*GC-NPFFR2* rs109452259	overdominant	0.4696	42439.3	CA	2.537	0.081
SCS	*BRCA1 rs134817801*	additive	0.1017	42438.9	AA	2.464	0.1
SCS	*BRCA1 rs134817801*	additive	0.1017	42438.9	AC	2.675	0.083
SCS	*BRCA1 rs134817801*	additive	0.1017	42438.9	CC	2.525	0.135
SCS	*BRCA1 rs134817801*	dominant	0.1603	42437.8	AA	2.463	0.1
SCS	*BRCA1 rs134817801*	dominant	0.1603	42437.8	AC+CC	2.634	0.071
SCS	*BRCA1 rs134817801*	recessive	0.6705	42439.6	AA+AC	2.589	0.065
SCS	*BRCA1 rs134817801*	recessive	0.6705	42439.6	CC	2.525	0.136
SCS	*BRCA1 rs134817801*	overdominant	0.0986	42437.1	AA+CC	2.486	0.08
SCS	*BRCA1 rs134817801*	overdominant	0.0986	42437.1	AC	2.676	0.083
SCS	*DGAT1* K232A	additive	0.5922	42437.4	KK	2.313	0.261
SCS	*DGAT1* K232A	additive	0.5922	42437.4	KA	2.461	0.092
SCS	*DGAT1* K232A	additive	0.5922	42437.4	AA	2.678	0.076
SCS	*DGAT1* K232A	dominant	0.3043	42438.7	KK	2.313	0.263
SCS	*DGAT1* K232A	dominant	0.3043	42438.7	KA+AA	2.59	0.06
SCS	*DGAT1* K232A	recessive	0.04397	42435.7	KK+KA	2.445	0.087
SCS	*DGAT1* K232A	recessive	0.04397	42435.7	AA	2.678	0.076
SCS	*DGAT1* K232A	overdominant	0.1102	42437.2	KK+AA	2.65	0.074
SCS	*DGAT1* K232A	overdominant	0.1102	42437.2	KA	2.462	0.092
MilkYield	*GC-NPFFR2* rs137147462	additive	0.9289	69860.1	AA	31.614	0.548
MilkYield	*GC-NPFFR2* rs137147462	additive	0.9289	69860.1	AG	31.557	0.329
MilkYield	*GC-NPFFR2* rs137147462	additive	0.9289	69860.1	GG	31.703	0.391
MilkYield	*GC-NPFFR2* rs137147462	dominant	0.9966	69858.1	AA	31.615	0.548
MilkYield	*GC-NPFFR2* rs137147462	dominant	0.9966	69858.1	AG+GG	31.618	0.252
MilkYield	*GC-NPFFR2* rs137147462	recessive	0.7863	69858.1	AA+AG	31.572	0.284
MilkYield	*GC-NPFFR2* rs137147462	recessive	0.7863	69858.1	GG	31.703	0.391
MilkYield	*GC-NPFFR2* rs137147462	overdominant	0.8004	69858.1	AA+GG	31.673	0.319
MilkYield	*GC-NPFFR2* rs137147462	overdominant	0.8004	69858.1	AG	31.558	0.329
MilkYield	*GC-NPFFR2* rs109452259	additive	0.1822	69856.1	CC	30.844	0.496
MilkYield	*GC-NPFFR2* rs109452259	additive	0.1822	69856.1	CA	31.626	0.317

MilkYield	*GC-NPFFR2* rs109452259	additive	0.1822	69856.1	AA	32.161	0.429
MilkYield	*GC-NPFFR2* rs109452259	dominant	0.08302	69855.1	CC	30.847	0.497
MilkYield	*GC-NPFFR2* rs109452259	dominant	0.08302	69855.1	CA+AA	31.815	0.256
MilkYield	*GC-NPFFR2* rs109452259	recessive	0.1352	69855.9	CC+CA	31.403	0.27
MilkYield	*GC-NPFFR2* rs109452259	recessive	0.1352	69855.9	AA	32.162	0.43
MilkYield	*GC-NPFFR2* rs109452259	overdominant	0.9427	69858.1	CC+AA	31.6	0.328
MilkYield	*GC-NPFFR2* rs109452259	overdominant	0.9427	69858.1	CA	31.633	0.32
MilkYield	*BRCA1 rs134817801*	additive	0.5624	69858	AA	31.939	0.394
MilkYield	*BRCA1 rs134817801*	additive	0.5624	69858	AC	31.644	0.329
MilkYield	*BRCA1 rs134817801*	additive	0.5624	69858	CC	30.96	0.536
MilkYield	*BRCA1 rs134817801*	dominant	0.321	69857.1	AA	31.936	0.395
MilkYield	*BRCA1 rs134817801*	dominant	0.321	69857.1	AC+CC	31.457	0.281
MilkYield	*BRCA1 rs134817801*	recessive	0.1768	69856.3	AA+AC	31.765	0.254
MilkYield	*BRCA1 rs134817801*	recessive	0.1768	69856.3	CC	30.961	0.536
MilkYield	*BRCA1 rs134817801*	overdominant	0.9229	69858.1	AA+CC	31.596	0.318
MilkYield	*BRCA1 rs134817801*	overdominant	0.9229	69858.1	AC	31.64	0.33
MilkYield	*DGAT1* K232A	additive	0.004487	69840.9	KK	28.047	1
MilkYield	*DGAT1* K232A	additive	0.004487	69840.9	KA	31.079	0.353
MilkYield	*DGAT1* K232A	additive	0.004487	69840.9	AA	32.263	0.292
MilkYield	*DGAT1* K232A	dominant	3.90E-04	69845.6	KK	28.058	1.014
MilkYield	*DGAT1* K232A	dominant	3.90E-04	69845.6	KA+AA	31.785	0.23
MilkYield	*DGAT1* K232A	recessive	8.47E-04	69847	KK+KA	30.754	0.34
MilkYield	*DGAT1* K232A	recessive	8.47E-04	69847	AA	32.269	0.297
MilkYield	*DGAT1* K232A	overdominant	0.0678	69854.8	KK+AA	31.945	0.291
MilkYield	*DGAT1* K232A	overdominant	0.0678	69854.8	KA	31.094	0.365
Fat %	*GC-NPFFR2* rs137147462	additive	0.924	16883	AA	3.912	0.05
Fat %	*GC-NPFFR2* rs137147462	additive	0.924	16883	AG	3.918	0.03
Fat %	*GC-NPFFR2* rs137147462	additive	0.924	16883	GG	3.905	0.036
Fat %	*GC-NPFFR2* rs137147462	dominant	0.9957	16881.1	AA	3.912	0.05
Fat %	*GC-NPFFR2* rs137147462	dominant	0.9957	16881.1	AG+GG	3.912	0.023
Fat %	*GC-NPFFR2* rs137147462	recessive	0.7959	16881	AA+AG	3.916	0.026
Fat %	*GC-NPFFR2* rs137147462	recessive	0.7959	16881	GG	3.905	0.036
Fat %	*GC-NPFFR2* rs137147462	overdominant	0.8037	16881	AA+GG	3.907	0.029
Fat %	*GC-NPFFR2* rs137147462	overdominant	0.8037	16881	AG	3.918	0.03
Fat %	*GC-NPFFR2* rs109452259	additive	0.7049	16882.6	CC	3.905	0.046
Fat %	*GC-NPFFR2* rs109452259	additive	0.7049	16882.6	CA	3.926	0.029
Fat %	*GC-NPFFR2* rs109452259	additive	0.7049	16882.6	AA	3.893	0.04
Fat %	*GC-NPFFR2* rs109452259	dominant	0.8583	16881.1	CC	3.905	0.046

Fat %	*GC-NPFFR2* rs109452259	dominant	0.8583	16881.1	CA+AA	3.914	0.024
Fat %	*GC-NPFFR2* rs109452259	recessive	0.5684	16880.8	CC+CA	3.92	0.025
Fat %	*GC-NPFFR2* rs109452259	recessive	0.5684	16880.8	AA	3.893	0.04
Fat %	*GC-NPFFR2* rs109452259	overdominant	0.5124	16880.7	CC+AA	3.898	0.03
Fat %	*GC-NPFFR2* rs109452259	overdominant	0.5124	16880.7	CA	3.925	0.029
Fat %	*BRCA1 rs134817801*	additive	0.1481	16876	AA	3.929	0.036
Fat %	*BRCA1 rs134817801*	additive	0.1481	16876	AC	3.862	0.03
Fat %	*BRCA1 rs134817801*	additive	0.1481	16876	CC	4.011	0.049
Fat %	*BRCA1 rs134817801*	dominant	0.5411	16880.7	AA	3.93	0.036
Fat %	*BRCA1 rs134817801*	dominant	0.5411	16880.7	AC+CC	3.903	0.026
Fat %	*BRCA1 rs134817801*	recessive	0.02497	16876.1	AA+AC	3.89	0.023
Fat %	*BRCA1 rs134817801*	recessive	0.02497	16876.1	CC	4.012	0.049
Fat %	*BRCA1 rs134817801*	overdominant	0.02168	16875.8	AA+CC	3.958	0.029
Fat %	*BRCA1 rs134817801*	overdominant	0.02168	16875.8	AC	3.863	0.03
Fat %	*DGAT1* K232A	additive	2.38E-07	16742.6	KK	4.507	0.073
Fat %	*DGAT1* K232A	additive	2.38E-07	16742.6	KA	4.095	0.026
Fat %	*DGAT1* K232A	additive	2.38E-07	16742.6	AA	3.736	0.021
Fat %	*DGAT1* K232A	dominant	2.65E-11	16836.5	KK	4.508	0.088
Fat %	*DGAT1* K232A	dominant	2.65E-11	16836.5	KA+AA	3.883	0.02
Fat %	*DGAT1* K232A	recessive	1.96E-26	16767.4	KK+KA	4.14	0.026
Fat %	*DGAT1* K232A	recessive	1.96E-26	16767.4	AA	3.735	0.023
Fat %	*DGAT1* K232A	overdominant	2.40E-13	16827.4	KK+AA	3.796	0.024
Fat %	*DGAT1* K232A	overdominant	2.40E-13	16827.4	KA	4.094	0.03
Protein %	*GC-NPFFR2* rs137147462	additive	0.3024	-3124	AA	3.477	0.03
Protein %	*GC-NPFFR2* rs137147462	additive	0.3024	-3124	AG	3.44	0.018
Protein %	*GC-NPFFR2* rs137147462	additive	0.3024	-3124	GG	3.408	0.022
Protein %	*GC-NPFFR2* rs137147462	dominant	0.1358	-3124.7	AA	3.477	0.03
Protein %	*GC-NPFFR2* rs137147462	dominant	0.1358	-3124.7	AG+GG	3.427	0.014
Protein %	*GC-NPFFR2* rs137147462	recessive	0.1145	-3125	AA+AG	3.45	0.016
Protein %	*GC-NPFFR2* rs137147462	recessive	0.1145	-3125	GG	3.408	0.022
Protein %	*GC-NPFFR2* rs137147462	overdominant	0.7222	-3122.6	AA+GG	3.431	0.018
Protein %	*GC-NPFFR2* rs137147462	overdominant	0.7222	-3122.6	AG	3.44	0.018
Protein %	*GC-NPFFR2* rs109452259	additive	0.6353	-3122.2	CC	3.434	0.028
Protein %	*GC-NPFFR2* rs109452259	additive	0.6353	-3122.2	CA	3.45	0.018
Protein %	*GC-NPFFR2* rs109452259	additive	0.6353	-3122.2	AA	3.411	0.024
Protein %	*GC-NPFFR2* rs109452259	dominant	0.9497	-3122.5	CC	3.434	0.028
Protein %	*GC-NPFFR2* rs109452259	dominant	0.9497	-3122.5	CA+AA	3.436	0.014
Protein %	*GC-NPFFR2* rs109452259	recessive	0.225	-3123.9	CC+CA	3.445	0.015

Protein %	*GC-NPFFR2* rs109452259	recessive	0.225	-3123.9	AA	3.411	0.024
Protein %	*GC-NPFFR2* rs109452259	overdominant	0.2559	-3123.8	CC+AA	3.421	0.018
Protein %	*GC-NPFFR2* rs109452259	overdominant	0.2559	-3123.8	CA	3.45	0.018
Protein %	*BRCA1 rs134817801*	additive	0.04777	-3125.8	AA	3.461	0.022
Protein %	*BRCA1 rs134817801*	additive	0.04777	-3125.8	AC	3.405	0.018
Protein %	*BRCA1 rs134817801*	additive	0.04777	-3125.8	CC	3.467	0.03
Protein %	*BRCA1 rs134817801*	dominant	0.1426	-3124.6	AA	3.462	0.022
Protein %	*BRCA1 rs134817801*	dominant	0.1426	-3124.6	AC+CC	3.422	0.016
Protein %	*BRCA1 rs134817801*	recessive	0.2402	-3123.8	AA+AC	3.429	0.014
Protein %	*BRCA1 rs134817801*	recessive	0.2402	-3123.8	CC	3.467	0.03
Protein %	*BRCA1 rs134817801*	overdominant	0.02167	-3127.8	AA+CC	3.463	0.017
Protein %	*BRCA1 rs134817801*	overdominant	0.02167	-3127.8	AC	3.406	0.018
Protein %	*DGAT1* K232A	additive	0.0585	-3178.3	KK	3.629	0.052
Protein %	*DGAT1* K232A	additive	0.0585	-3178.3	KA	3.525	0.018
Protein %	*DGAT1* K232A	additive	0.0585	-3178.3	AA	3.357	0.015
Protein %	*DGAT1* K232A	dominant	0.000494	-3134.7	KK	3.63	0.056
Protein %	*DGAT1* K232A	dominant	0.000494	-3134.7	KA+AA	3.426	0.013
Protein %	*DGAT1* K232A	recessive	2.00E-13	-3176.7	KK+KA	3.536	0.017
Protein %	*DGAT1* K232A	recessive	2.00E-13	-3176.7	AA	3.357	0.015
Protein %	*DGAT1* K232A	overdominant	7.33E-09	-3156	KK+AA	3.379	0.015
Protein %	*DGAT1* K232A	overdominant	7.33E-09	-3156	KA	3.525	0.019
SNF %	*GC-NPFFR2* rs137147462	additive	0.6122	194	AA	8.956	0.037
SNF %	*GC-NPFFR2* rs137147462	additive	0.6122	194	AG	8.934	0.022
SNF %	*GC-NPFFR2* rs137147462	additive	0.6122	194	GG	8.86	0.027
SNF %	*GC-NPFFR2* rs137147462	dominant	0.2022	196.6	AA	8.955	0.037
SNF %	*GC-NPFFR2* rs137147462	dominant	0.2022	196.6	AG+GG	8.903	0.017
SNF %	*GC-NPFFR2* rs137147462	recessive	0.0151	192.3	AA+AG	8.94	0.019
SNF %	*GC-NPFFR2* rs137147462	recessive	0.0151	192.3	GG	8.86	0.027
SNF %	*GC-NPFFR2* rs137147462	overdominant	0.1868	196.5	AA+GG	8.892	0.022
SNF %	*GC-NPFFR2* rs137147462	overdominant	0.1868	196.5	AG	8.934	0.022
SNF %	*GC-NPFFR2* rs109452259	additive	0.4028	195.3	CC	8.908	0.034
SNF %	*GC-NPFFR2* rs109452259	additive	0.4028	195.3	CA	8.942	0.022
SNF %	*GC-NPFFR2* rs109452259	additive	0.4028	195.3	AA	8.861	0.029
SNF %	*GC-NPFFR2* rs109452259	dominant	0.8874	198.2	CC	8.908	0.034
SNF %	*GC-NPFFR2* rs109452259	dominant	0.8874	198.2	CA+AA	8.913	0.018
SNF %	*GC-NPFFR2* rs109452259	recessive	0.04188	194	CC+CA	8.932	0.018
SNF %	*GC-NPFFR2* rs109452259	recessive	0.04188	194	AA	8.861	0.03
SNF %	*GC-NPFFR2* rs109452259	overdominant	0.053	194.4	CC+AA	8.881	0.022

SNF %	*GC-NPFFR2* rs109452259	overdominant	0.053	194.4	CA	8.942	0.022
SNF %	*BRCA1 rs134817801*	additive	0.05351	195.1	AA	8.943	0.027
SNF %	*BRCA1 rs134817801*	additive	0.05351	195.1	AC	8.876	0.022
SNF %	*BRCA1 rs134817801*	additive	0.05351	195.1	CC	8.952	0.037
SNF %	*BRCA1 rs134817801*	dominant	0.1559	196.2	AA	8.943	0.027
SNF %	*BRCA1 rs134817801*	dominant	0.1559	196.2	AC+CC	8.896	0.019
SNF %	*BRCA1 rs134817801*	recessive	0.2383	196.8	AA+AC	8.904	0.017
SNF %	*BRCA1 rs134817801*	recessive	0.2383	196.8	CC	8.952	0.037
SNF %	*BRCA1 rs134817801*	overdominant	0.02418	193.1	AA+CC	8.946	0.022
SNF %	*BRCA1 rs134817801*	overdominant	0.02418	193.1	AC	8.876	0.022
SNF %	*DGAT1* K232A	additive	0.08364	148.7	KK	9.136	0.065
SNF %	*DGAT1* K232A	additive	0.08364	148.7	KA	9.017	0.023
SNF %	*DGAT1* K232A	additive	0.08364	148.7	AA	8.821	0.019
SNF %	*DGAT1* K232A	dominant	0.001092	187.5	KK	9.137	0.07
SNF %	*DGAT1* K232A	dominant	0.001092	187.5	KA+AA	8.901	0.016
SNF %	*DGAT1* K232A	recessive	3.76E-12	149.7	KK+KA	9.03	0.022
SNF %	*DGAT1* K232A	recessive	3.76E-12	149.7	AA	8.821	0.019
SNF %	*DGAT1* K232A	overdominant	3.81E-08	167.9	KK+AA	8.846	0.019
SNF %	*DGAT1* K232A	overdominant	3.81E-08	167.9	KA	9.017	0.024
